# VITAL: Value-Invariant Transformation and Alignment Learning for quantitative photoacoustic microscopy^☆^^☆☆^

**DOI:** 10.1016/j.pacs.2026.100845

**Published:** 2026-06-08

**Authors:** Shuocheng Qi, Mingxuan Wang, Yachao Zhang, Lidai Wang, Chao Liu

**Affiliations:** a Digital Medical Research Center, School of Basic Medical Sciences, Fudan University, Shanghai, China; b Shanghai Key Laboratory of Medical Imaging Computing and Computer Assisted Intervention, Shanghai, China; c School of Intelligent Equipment, Shandong University of Science and Technology, Shandong, China; d Department of Biomedical Engineering, City University of Hong Kong, Kowloon, Hong Kong Special Administrative Region of China; e Shenzhen Research Institute, City University of Hong Kong, Shenzhen, China; f School of Information Science and Technology, Guangdong University of Foreign Studies, Guangzhou, China; g The Suzhou Institute of Biomedical Engineering and Technology, Chinese Academy of Sciences, Suzhou 215163, China; h The School of Biomedical Engineering (Suzhou), Division of Life Sciences and Medicine, University of Science and Technology of China, Hefei 230026, China

**Keywords:** Image registration, Keypoint matching, Thin-plate spline, Nearest-neighbor warping, Blood oxygen saturation, Photoacoustic microscopy

## Abstract

Fast optical-resolution photoacoustic microscopy (OR-PAM) enables dynamic microvascular imaging but suffers from spatial misalignment and biased functional quantification that are inadequately solved by conventional registration methods. We propose VITAL (Value-Invariant Transformation and Alignment Learning), a cascaded PA signal amplitude-preserving (PSAP) registration framework that separates geometric alignment from preservation of discrete PA signal-amplitude distributions. VITAL combines interpretable keypoint-based global alignment using SuperPoint, LightGlue, and thin-plate spline transformation with topology-preserving dense refinement for sub-pixel accuracy. To reduce interpolation-induced signal distortion, nearest-neighbor warping is adopted to avoid interpolation-generated PA signal amplitudes. Validated on *in vivo* mouse brain imaging under oxygen challenge and controlled synthetic deformation, fine-tuned VITAL achieves an 89.1% reduction in mean squared error (MSE), improves normalized cross-correlation (NCC) from 0.178 to 0.821, and maintains a PA signal-amplitude distribution fidelity rate (PFr) of 97.83%, supporting robust downstream functional OR-PAM analysis.

## 1 Introduction

Quantitative analysis of microvascular function is essential for understanding physiological and pathological processes, yet remains challenging in high-speed biomedical imaging [1], [2], [3]. In optical-resolution photoacoustic microscopy (OR-PAM), subcellular resolution combined with intrinsic hemoglobin contrast allows direct observation of microvascular hemodynamics, blood flow dynamics, and oxygen metabolism *in vivo* [4], [5], [6]. These capabilities establish OR-PAM as a powerful tool for quantitative vascular imaging in applications such as cerebral perfusion, tumor vascular remodeling, and neurovascular coupling [7], [8], [9], [10].

Capturing these dynamic processes requires imaging systems capable of high temporal resolution [11], [12]. To increase imaging speed, modern OR-PAM systems employ fast scanning strategies such as microelectromechanical system (MEMS) scanners [3], voice-coil scanners [6], rotary scanners [9], and polygon mirrors [7], [8]. However, like other high-speed imaging modalities [13], [14], [15], fast OR-PAM (e.g., voice-coil-driven) introduces coupled distortions arising from both intra-frame and inter-frame dynamics. Within a single frame, scan trajectory nonlinearity and temporal sampling asymmetry lead to inconsistencies between forward and backward scan lines. Across frames, long-term mechanical hysteresis and actuator instability gradually change the scanning trajectory, leading to inter-frame misalignment that accumulates over time. Although arising from distinct physical mechanisms, these effects are fundamentally coupled and jointly manifest as two core challenges: (1) geometric distortion leading to spatial misalignment within and across frames, and (2) functional quantification bias induced by alterations of original PA signal amplitudes during conventional image registration.

To correct spatial misalignment, many conventional registration methods, such as Scale-Invariant Feature Transform (SIFT) [16], optical flow [17], Demons [18], and Symmetric Normalization (SyN) [19], have been widely used. However, these methods rely on the assumption of signal consistency, which is not satisfied in bidirectional OR-PAM due to acquisition-induced PA amplitude variation, leading to degraded performance. Recently, learning-based registration methods, including VoxelMorph [20], TransMorph [21], and affine-deformable frameworks [22], have been explored for image registration [23], [24]. These methods commonly optimize image-similarity losses such as NCC together with deformation regularization, and therefore still rely, at least implicitly, on consistency between corresponding PA signal amplitudes. To address this limitation, an alternative strategy is to perform registration in feature space rather than relying on raw signal-intensity comparisons, because feature-based matching is less sensitive to acquisition-induced PA amplitude variation. For instance, SuperPoint [25] provides self-supervised keypoint detection with discriminative descriptors, while LightGlue [26] enables transformer-based matching. Keypoint-driven registration has shown promise in several biomedical imaging applications, including brain image registration with KeyMorph [27], spatial transcriptomics alignment using thin-plate spline (TPS) models with learned landmarks [28], and histological image alignment through iterative methods [29]. However, such approaches remain largely unexplored for vascular registration in OR-PAM, where thin vessels, bifurcations, and crossings present unique structural challenges.

To reduce functional quantification bias, registration should avoid generating PA signal amplitudes that were not directly measured. In quantitative imaging, functional parameters such as oxygen saturation (sO${}_{2}$), blood flow speeds ($v_{\text{flow}}$), and vascular continuity are derived directly from PA signal amplitudes [1], [4], [8]. Conventional registration pipelines typically rely on interpolation-based warping to resample images after geometric transformation. However, interpolation changes the original PA signal amplitudes, potentially introducing bias into downstream functional quantification [4], [8]. Therefore, registration methods should not only achieve accurate geometric alignment but also provide sufficient interpretability to verify geometric correspondence and to track whether registered PA signal amplitudes originate from measured source PA samples rather than interpolation. Dense deformation fields predicted by learning-based methods such as VoxelMorph or TransMorph are often difficult to interpret, making it challenging to identify the origin of potential misregistration errors. In contrast, keypoint-based registration offers improved interpretability in addition to robustness. Explicit keypoint correspondences provide a transparent mechanism for assessing registration quality: each matched keypoint pair can be directly visualized relative to the underlying vascular anatomy, enabling verification prior to functional analysis. This transparency helps reduce the risk of error propagation into hemodynamic measurements and improves the reliability of quantitative functional estimation.

To address this challenge, we propose VITAL (Value-Invariant Transformation and Alignment Learning), a cascaded PA signal amplitude-preserving (PSAP) registration framework that explicitly decouples geometric alignment from preservation of discrete source PA amplitude distributions. First, an interpretable global alignment is achieved using SuperPoint-based keypoint detection, LightGlue-based matching, and TPS transformation, enabling robust geometric correction under PA amplitude inconsistency. Second, residual sub-pixel misalignment is refined by predicting dense deformation fields with topology-preserving regularization. A per-pair fine-tuning step is applied during inference to further optimize the deformation field. To avoid interpolation-generated PA signal amplitudes, nearest-neighbor warping is employed as the final resampling step, improving quantitative reliability by retaining measured source PA amplitudes and thereby supporting preservation of discrete source PA amplitude distributions. The proposed method is validated on *in vivo* mouse brain imaging via a three-wavelength (532, 545, and 558 nm) OR-PAM system and a controlled synthetic deformation dataset, with both direct application and per-pair fine-tuning evaluated. VITAL outperforms six representative baseline methods across six evaluation metrics, with fine-tuned VITAL achieving an 89.1% reduction in mean squared error (MSE) and improving normalized cross-correlation (NCC) from 0.178 to 0.821 on the real held-out Dataset I (DI). These results reduce interpolation-induced signal distortion in downstream functional analysis, including sO${}_{2}$, blood-flow-speed estimation, and vascular-continuity assessment under an oxygen challenge experiment. Together, these results demonstrate the importance of PSAP registration for quantitative functional OR-PAM.

## 2 Methods

**Fig. 1:**
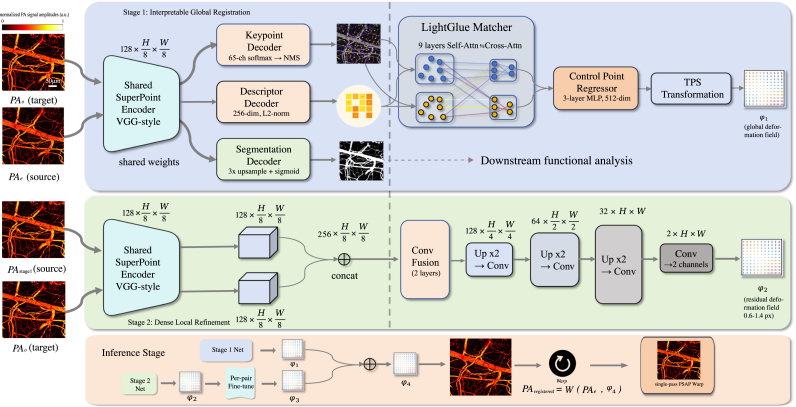
Overall architecture of the proposed VITAL framework. Stage 1 performs interpretable global registration via SuperPoint feature extraction, LightGlue matching, and thin-plate spline transformation, producing global deformation field $\phi_{1}$. Stage 2 refines residual sub-pixel misalignment through a shared-encoder UNet predicting dense deformation field $\phi_{2}$. A per-pair fine-tuning step optimizes $\phi_{2}$ into $\phi_{3}$, which is composed with $\phi_{1}$ to yield $\phi_{4}$. The source image is warped once using $\phi_{4}$ with nearest-neighbor warping.

Fig. 1 introduces the overall design of VITAL. Both stages share a SuperPoint-based VGG-style encoder, but they have different functions. Stage 1 detects and matches vascular keypoints and then fits a TPS transformation to produce the coarse deformation field $\phi_{1}$. Stage 2 keeps the shared encoder, fuses source and target features, and predicts a dense residual deformation field $\phi_{2}$ with a UNet decoder. At test time, $\phi_{2}$ is further refined on each image pair to obtain $\phi_{3}$, which is composed with $\phi_{1}$ before a single nearest-neighbor warp is applied. This decomposition is deliberate, ensuring that large-scale corrections remain interpretable while small-scale refinements are appropriately constrained.

### 2.1 Problem formulation

**Fig. 2:**
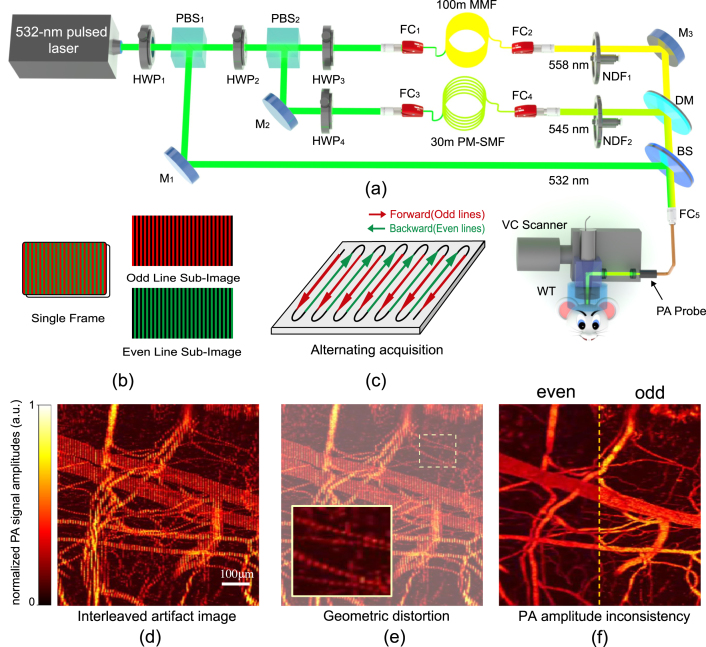
Schematic of a high-speed three-wavelength OR-PAM system and the associated registration challenges. (a) 3D schematic of the OR-PAM optical system. BS: beam splitter (R/T: 10/90), DM: dichroic mirror (550 nm long-pass), FC1–5: fiber coupler, HWP1–4: half-wave plate, M1–3: mirror, MMF: multi-mode fiber, PBS1–2: polarizing beam splitter, PM-SMF: polarization-maintaining single-mode fiber, NDF1–2: neutral density filter, VC scanner: voice-coil scanner, WT: water tank. (b) Odd-line and even-line sub-image extraction from a single frame. (c) Voice-coil raster scanning with alternating forward/backward lines. (d) Interleaved image showing stripe artifacts from coupled geometric distortion and PA amplitude inconsistency. (e) Geometric distortion in a local vascular region. (f) PA amplitude inconsistency between even and odd line images.

Fig. 2a illustrates the multi-wavelength OR-PAM system. Fig. 2b–f demonstrate how bidirectional scanning decomposes a single acquisition into odd and even sub-images with coupled geometric distortion and amplitude inconsistency. Importantly, this visualization reveals that the registration challenge arises intrinsically during data acquisition rather than in downstream reconstruction.

As shown in Fig. 2b–c, each frame is acquired with alternating forward and backward raster lines. The acquisition can be decomposed into two half-resolution PA amplitude images, $PA_{o}$ and $PA_{e}$, corresponding to forward and backward scans, forming the odd- and even-line images, respectively. Both images encode the same underlying vascular morphological characteristics C, but exhibit systematic discrepancies induced by direction-dependent acquisition conditions. The measurement model is

(1) $$PA\left(x,y,\tau ,d\right)=H\;\left(C\left(x,y,\tau \right),\;\theta_{d}\right)+\epsilon$$

where (x,y) are spatial coordinates, τ is the acquisition time, and d∈{fwd,bwd} denotes scan direction. The latent vascular anatomy is C(x,y,τ), H is the direction-dependent image formation operator, $\theta_{d}$ collects direction-dependent acquisition factors, and ϵ denotes measurement noise. Because $\theta_{\text{fwd}}\neq \theta_{\text{bwd}}$, the same anatomical structure is mapped to different signal amplitudes in the two scan directions, thereby violating the signal consistency assumption. Accordingly, $PA_{o}$ is treated as the fixed image and $PA_{e}$ as the moving image, and ϕ is estimated in a learned feature space that is less sensitive to PA amplitude inconsistency. This formulation is not only descriptive. It clarifies why geometric and amplitude components must be separated rather than jointly optimized.

For inter-frame registration, given a temporal sequence ${\left\{PA^{\left(t\right)}\right\}}_{t=1}^{T}$ of artifact-corrected frames, spatial mappings $\left\{\phi^{\left(t\right)}\right\}$ are estimated to align every frame to a common reference $PA^{\left(1\right)}$. This establishes a unified coordinate system for the entire sequence, enabling longitudinal functional analysis.

The main challenge arises when $\theta_{\text{fwd}}\neq \theta_{\text{bwd}}$, causing odd and even scan lines to exhibit amplitude discrepancies even when imaging the same vessel. As shown in Fig. 2d–f, interleaving the two line sets produces stripe artifacts at every odd–even boundary, arising from a combination of spatial misalignment and amplitude inconsistency. VITAL avoids forcing these two effects into one signal-intensity-based objective by matching learned keypoint descriptors that remain stable under scan-direction-dependent amplitude variation. This decoupling establishes the core conceptual innovation of the method.

### 2.2 Stage 1: Interpretable global registration

Stage 1 is where most of the interpretable correction happens. It extracts shared features, detects keypoints, matches them with a transformer, and fits a TPS transformation. Each intermediate result can be directly inspected, which is important because the largest registration errors typically originate at this coarse stage.

#### 2.2.1 Shared feature encoder (SuperPoint)

The shared backbone is a SuperPoint-based encoder [25] with a VGG-style architecture. Four convolutional blocks and three max-pooling layers reduce the input to a 1/8-resolution feature map:

(2) $$F=E_{\text{SP}}\left(PA\right)\in R^{128\times H/8\times W/8}$$

Source and target images pass through the same encoder. In practice, this encourages the features to follow vessel morphology rather than scan-direction-specific amplitude changes.

#### 2.2.2 Keypoint detection and description

Two decoder heads read from the shared features F: one predicts keypoints and the other predicts descriptors.

The keypoint head produces a dense score map through a 65-channel softmax output (64 spatial bins + 1 dustbin). The map is then reshaped to full resolution and non-maximum suppression (NMS) is applied with radius r=4:

(3) $${\left\{\left(p_{i},s_{i}\right)\right\}}_{i=1}^{N}=D_{\text{kp}}\left(F\right),\;p_{i}\in R^{2},\;s_{i}\in \left[0,1\right]$$

where $p_{i}$ and $s_{i}$ are the location and confidence of the ith keypoint. The detection threshold is set to $\tau_{\text{det}}=0.005$, and the total number of keypoints is capped at $N_{\max}=4096$.

The descriptor head samples a 256-dimensional L2-normalized feature vector at each keypoint location from a learned descriptor map:

(4) $$d_{i}=D_{\text{desc}}\left(F,p_{i}\right)\in R^{256},\;{\left\|d_{i}\right\|}_{2}=1$$

These descriptors mainly encode local vessel geometry, which makes matching less sensitive to scan-direction-dependent amplitude differences.

#### 2.2.3 Learned vessel mask

In addition to keypoints, the shared encoder also feeds a lightweight segmentation decoder that predicts a vessel mask:

(5) $$M=D_{\text{seg}}\left(F\right)\in {\left[0,1\right]}^{H\times W}$$

The decoder uses three upsampling stages with batch normalization and a sigmoid output. For supervision, we generate pseudo-labels with Otsu thresholding [30] on the target image, so no manual vessel annotation is required. The decoder produces a soft vessel probability map that serves as an auxiliary supervision signal and encourages the shared encoder to focus on vascular structures. This predicted soft mask is used to restrict downstream functional analysis, including sO${}_{2}$ mapping and $v_{\text{flow}}$ estimation, to vascular regions. Importantly, it is not used as the region of interest (ROI) for quantitative registration evaluation. For all reported registration metrics, the evaluation ROI is a method-independent binary vessel mask generated from the fixed/target image using Otsu thresholding and identical post-processing. The same Otsu-derived ROI is used for VITAL and all baseline methods.

#### 2.2.4 Feature matching (LightGlue)

We match source and target keypoints with LightGlue [26], a transformer-based matcher with nine alternating self-attention and cross-attention layers:

(6) $$\text{Self-Attn}:\;d_{i}^{\left(l\right)}=d_{i}^{\left(l-1\right)}+\text{MHA}\left(d_{i}^{\left(l-1\right)},{\left\{d_{j}^{\left(l-1\right)}\right\}}_{j\in \text{same}}\right)$$

(7) $$\text{Cross-Attn}:\;d_{i}^{\left(l\right)}=d_{i}^{\left(l\right)}+\text{MHA}\left(d_{i}^{\left(l\right)},{\left\{d_{j}^{\left(l\right)}\right\}}_{j\in \text{other}}\right)$$

Here, MHA denotes multi-head attention. Self-attention models spatial relationships within each image, while cross-attention links source and target features. The output is a set of matched keypoint pairs ${\left\{\left(p_{i}^{\text{src}},p_{i}^{\text{tgt}}\right)\right\}}_{i=1}^{M}$ with confidence scores.

**Fig. 3:**
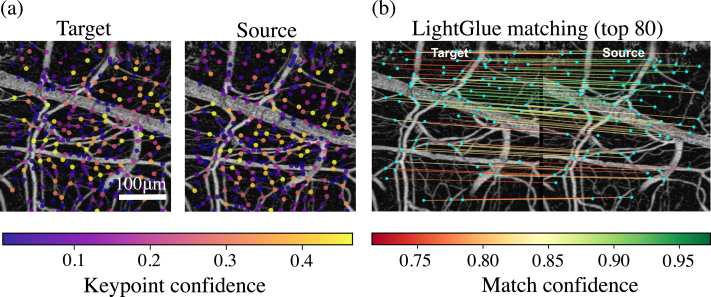
Keypoint detection and matching in Stage 1. (a) SuperPoint keypoints on target and source sub-images. (b) LightGlue matches.

These matches are directly interpretable. Fig. 3a shows that SuperPoint concentrates on vascular bifurcations, crossings, and endpoints. Fig. 3b shows the matched pairs colored by confidence. The value of this visualization is practical: it provides a direct way to evaluate whether the model aligns vessels based on appropriate structural cues.

#### 2.2.5 Control point regression and TPS transformation

Matched keypoint pairs act as control points for a TPS transformation. LightGlue gives reliable correspondences, but the coordinates still inherit sub-pixel localization error from the SuperPoint heatmap. To reduce that error, we add a learned control-point regressor, implemented as a 3-layer multilayer perceptron (MLP) with 512 hidden units, that takes the match coordinates and descriptor features and predicts refined 2D displacements:

(8) $$\Delta p_{i}=\text{MLP}\left(\left[p_{i}^{\text{src}},p_{i}^{\text{tgt}},d_{i}^{\text{src}},d_{i}^{\text{tgt}}\right]\right)\in R^{2}$$

We train this regressor end to end. Gradients from the downstream TPS warping loss ($L_{\text{MSE}}$ in Eq. (10)) propagate through the differentiable TPS layer to directly update the MLP, ensuring that the predicted corrections are driven by the final registration error rather than a separate proxy objective. This is important because small localization errors at the control-point stage would otherwise be propagated into the global warp. The TPS transformation [31] then turns these sparse displacements into a smooth dense deformation field $\phi_{1}\in R^{2\times H\times W}$ using the kernel $K\left(r\right)=r^{2}\log r$:

(9) $$\phi_{1}\left(x\right)=\overset{M}{\underset{i=1}{\sum }}w_{i}K\left(\|x-p_{i}^{\text{tgt}}\|\right)+a_{0}+a_{1}x$$

where $w_{i}$ are the TPS weights and $a_{0},a_{1}$ are affine parameters. The key point is that the global warp is entirely determined by explicit and directly observable correspondences. This enhances the interpretation of Stage 1 relative to purely dense black-box deformations, although ambiguity may still persist in challenging regions. The warped source image is written as $PA_{\text{stage1}}=PA_{e}\circ \phi_{1}^{-1}$.

#### 2.2.6 Stage 1 training objective

Stage 1 is trained with the objective

(10) $$L_{\text{total}}^{\text{S1}}=L_{\text{MSE}}+L_{\text{BCE}}+\lambda_{\text{def}}{\left\|\phi_{1}\right\|}_{2}+L_{\text{dnh}}$$

where $L_{\text{MSE}}$ is the reconstruction loss, $L_{\text{BCE}}$ (binary cross-entropy) supervises the segmentation decoder, and ${\left\|\phi_{1}\right\|}_{2}$ regularizes the deformation magnitude with $\lambda_{\text{def}}=10^{-5}$. A do-no-harm term is also included so the network is penalized only when registration makes the alignment worse than the unregistered input:

(11) $$L_{\text{dnh}}=\max\;(0,\;\text{MSE}\left(PA_{\text{warped}},PA_{\text{target}}\right)-\text{MSE}\left(PA_{\text{source}},PA_{\text{target}}\right))$$

This penalty is activated only when the warped image is less similar to the target than the original source image. In practice, it prevents Stage 1 from learning aggressive and harmful deformations. In Stage 1, SuperPoint–LightGlue matches provide explicit vascular correspondences, and TPS converts these sparse correspondences into a smooth global deformation field. The MSE term provides an end-to-end optimization signal for refining the matched control points through the differentiable TPS layer, while the do-no-harm term stabilizes training by suppressing transformations that reduce source–target similarity relative to the unregistered input. This design combines correspondence-driven global alignment with a bounded image-level refinement signal, while local residual deformation is handled separately by Stage 2.

### 2.3 Stage 2: Dense local refinement

Stage 1 corrects large misalignments, but small residual errors remain. Stage 2 addresses these sub-pixel differences with a dense deformation field, while smoothness and Jacobian constraints restrict the corrections to a narrow and physically plausible range. This narrow range is important, as it allows Stage 2 to refine alignment without reintroducing the larger ambiguities already resolved in Stage 1.

#### 2.3.1 Shared encoder feature extraction

The coarsely aligned image $PA_{\text{stage1}}$ and the target $PA_{o}$ are processed by a shared SuperPoint encoder, initialized from Stage 1, to extract features:

(12) $$F_{\text{fixed}}=E_{\text{SP}}\left(PA_{o}\right)\in R^{128\times H/8\times W/8}$$

(13) $$F_{\text{moving}}=E_{\text{SP}}\left(PA_{\text{stage1}}\right)\in R^{128\times H/8\times W/8}$$

We concatenate the two feature maps along the channel dimension and pass them through a two-layer fusion block:

(14) $$F_{\text{fused}}={\text{Conv}}_{\text{fusion}}\left(\left[F_{\text{fixed}};F_{\text{moving}}\right]\right)\in R^{256\times H/8\times W/8}$$

#### 2.3.2 UNet decoder for dense deformation prediction

A three-stage upsampling decoder restores spatial resolution and predicts the residual deformation field:

(15) $$D_{1}={\text{Conv}}_{128}\left({\text{Up}}_{\times 2}\left(F_{\text{fused}}\right)\right)\in R^{128\times H/4\times W/4}$$

(16) $$D_{2}={\text{Conv}}_{64}\left({\text{Up}}_{\times 2}\left(D_{1}\right)\right)\in R^{64\times H/2\times W/2}$$

(17) $$D_{3}={\text{Conv}}_{32}\left({\text{Up}}_{\times 2}\left(D_{2}\right)\right)\in R^{32\times H\times W}$$

(18) $$\phi_{2}={\text{Conv}}_{2}\left(D_{3}\right)\in R^{2\times H\times W}$$

Each decoder stage upsamples bilinearly and then applies two convolutional layers with rectified linear unit (ReLU) activation. The final 2-channel output is the residual deformation field $\phi_{2}$. During inference, per-pair fine-tuning (Section 2.5) updates $\phi_{2}$ to a refined field $\phi_{3}$, which is then composed with $\phi_{1}$:

(19) $$\phi_{4}=\phi_{3}\circ \phi_{1}$$

(20) $$PA_{\text{registered}}=\text{Warp}\left(PA_{e},\phi_{4}\right)$$

**Fig. 4:**
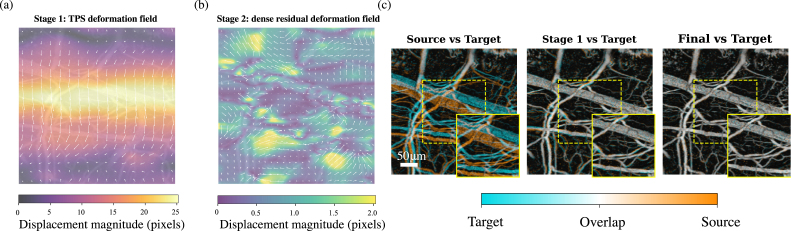
Visualization of deformation fields from the two-stage registration. (a) Stage 1 TPS deformation field showing large-scale global deformation (magnitude 5–20 pixels), with arrows indicating displacement direction and color encoding magnitude. (b) Stage 2 dense residual deformation field showing fine-scale sub-pixel refinement (magnitude 0.6–1.4 pixels). (c) Overlay of source, Stage 1 warped, and final registered images demonstrating progressive alignment improvement.

Stage 2 corrections are typically only 0.6–1.4 pixels, which confirms that Stage 1 already removes most of the geometric error. Fig. 4 makes this split visible: the TPS field captures the larger 5–20 pixel deformation, while the residual field cleans up the remaining local misalignment. This separation of scales also explains why the second stage can remain conservative without becoming ineffective.

### 2.4 Stage 2 loss functions

Stage 2 can rely on intensity-based similarity losses because it does not operate on the raw misaligned pair. By the time it is applied, Stage 1 has already removed the large misalignment and reduced the remaining error to approximately 0.6–1.4 pixels. At that scale, local alignment gradients are more informative than those produced by scan-direction amplitude differences. In other words, intensity-based terms are unstable at the initial stage of the problem but become effective after coarse alignment. The MSE term in Stage 1 serves solely as an auxiliary signal, with the TPS update remaining primarily driven by feature matching. This use of intensity terms is therefore confined to constrained residual refinement in OR-PAM.

The training objective for Stage 2 consists of similarity losses and regularization terms.

#### 2.4.1 Similarity losses

Once Stage 1 has reduced the large geometric error, Stage 2 refines the remaining misalignment with three complementary similarity terms based on Dice overlap, structural similarity index measure (SSIM), and NCC:

(21) $$L_{\text{sim}}=\lambda_{\text{dice}}L_{\text{dice}}+\lambda_{\text{SSIM}}L_{\text{SSIM}}+\lambda_{\text{NCC}}L_{\text{NCC}}$$

$L_{\text{dice}}$ measures overlap between warped and target vessel masks, $L_{\text{SSIM}}$ measures structural similarity [32], and $L_{\text{NCC}}$ captures local correspondence after coarse alignment. The weights are set to $\lambda_{\text{dice}}=0.3$, $\lambda_{\text{SSIM}}=0.1$, and $\lambda_{\text{NCC}}=0.3$. Global NCC performs well at this stage because the remaining effect of PA amplitude inconsistency is already small.

#### 2.4.2 Topology-preserving regularization

To ensure that the deformation remains anatomically reasonable, Stage 2 uses a composite regularization loss:

(22) $$L_{\text{reg}}=\lambda_{\text{smooth}}L_{\text{smooth}}+\lambda_{\text{jac}}L_{\text{jac}}$$

The smoothness term $L_{\text{smooth}}$ penalizes first- and second-order spatial gradients of the deformation field so that neighboring pixels move coherently:

(23) $$L_{\text{smooth}}=\frac{1}{4}\left({\left\|\nabla_{x}\phi \right\|}_{1}+{\left\|\nabla_{y}\phi \right\|}_{1}+{\left\|\nabla_{xx}\phi \right\|}_{1}+{\left\|\nabla_{yy}\phi \right\|}_{1}\right)$$

The Jacobian term $L_{\text{jac}}$ penalizes negative determinants and therefore discourages topological folding [33]:

(24) $$L_{\text{jac}}=\frac{1}{\left|\Omega \right|}\underset{x\in \Omega }{\sum }{\left[\max\left(0,-\det\left(J_{\phi }\left(x\right)\right)\right)\right]}^{2}$$

Here, $J_{\phi }\left(x\right)$ is the Jacobian matrix at location x. Positive determinants correspond to locally invertible mappings, which helps prevent vessel crossings or fold-overs. The regularization weights are $\lambda_{\text{smooth}}=0.2$ and $\lambda_{\text{jac}}=0.3$. This regularization serves more than a visual purpose; without it, a numerically accurate warp may still yield anatomically inconsistent vessel structures.

#### 2.4.3 Total training objective

The complete training loss for Stage 2 combines all terms:

(25) $$L_{\text{total}}^{\text{S2}}=L_{\text{sim}}+L_{\text{reg}}$$

### 2.5 Per-pair fine-tuning

Recent work in image registration indicates that a combination of learned initialization and instance-specific optimization outperforms either strategy alone [34], [35]. Similarly, the pretrained Stage 2 network provides an amortized initialization that captures deformation patterns learned from the training data. For each test pair, the Stage 2 decoder is then optimized for a fixed number of iterations (300 steps) using the same loss $L_{\text{total}}^{\text{S2}}$ (Eq. (25)), starting from $\phi_{2}$ and producing a refined field $\phi_{3}$. The Stage 1 encoder and $\phi_{1}$ stay fixed. The final field is $\phi_{4}=\phi_{3}\circ \phi_{1}$. This additional optimization step enables the model to adapt to pair-specific anatomical variations that are not fully captured during training, at the expense of increased inference time.

### 2.6 Nearest-neighbor warping for avoiding interpolation-generated PA amplitudes

In OR-PAM, PA signal amplitude arises from the optical absorption $\mu_{a}\left(\lambda \right)$:

(26) $$\text{PA}\left(\lambda \right)\propto \Gamma \cdot \eta_{\text{th}}\cdot \mu_{a}\left(\lambda \right)\cdot \Phi \left(\lambda \right)$$

where Γ is the Grüneisen parameter, $\eta_{\text{th}}$ is the thermal conversion efficiency, and Φ is the local fluence. For functional PA quantification, bilinear or bicubic interpolation mixes neighboring pixels and alters PA signal amplitudes, thereby biasing downstream functional estimates.

For the final warp, nearest-neighbor warping is used:

(27) $$PA_{\text{registered}}\left(x\right)=PA_{\text{source}}\left(\text{round}\left(\phi^{-1}\left(x\right)\right)\right)$$

This means every output pixel is copied from an existing uint16 PA amplitude in the source image rather than synthesized by interpolation, so the final registered image does not introduce new PA signal amplitudes. On the DI real OR-PAM test set, the fine-tuned VITAL model achieves a PA signal-amplitude distribution fidelity rate (PFr) of 97.83%. PFr does not need to reach exactly 100%, because spatial resampling can still redistribute the frequency of existing amplitudes even when no new amplitudes are created. In other words, nearest-neighbor warping avoids interpolation-generated PA signal amplitudes and retains measured source PA amplitudes, thereby supporting preservation of discrete source PA amplitude distributions rather than guaranteeing exact histogram equality.

During training, bilinear interpolation is still used because the optimization needs a differentiable sampler. At inference, however, the per-pair refined field $\phi_{3}$ is first composed with the Stage 1 field $\phi_{1}$ to obtain a single field $\phi_{4}=\phi_{3}\circ \phi_{1}$, and the source image is then warped only once with nearest-neighbor warping. This avoids the extra rounding loss that would come from repeated resampling.

Avoiding new PA signal amplitudes depends on two factors. First, nearest-neighbor warping determines how measured source PA amplitudes are sampled, ensuring that each output pixel is drawn directly from the original source grid. Second, Stage 1 keypoint matching makes the coarse warp partially interpretable and therefore indicates where those measured source amplitudes are sampled. Large mapping errors can be identified relative to vascular anatomy rather than being hidden within an opaque dense deformation field. The combination is critical because accurate sampling is as important as avoiding interpolation-generated amplitudes.

### 2.7 Intra-frame and inter-frame registration pipeline

**Fig. 5:**
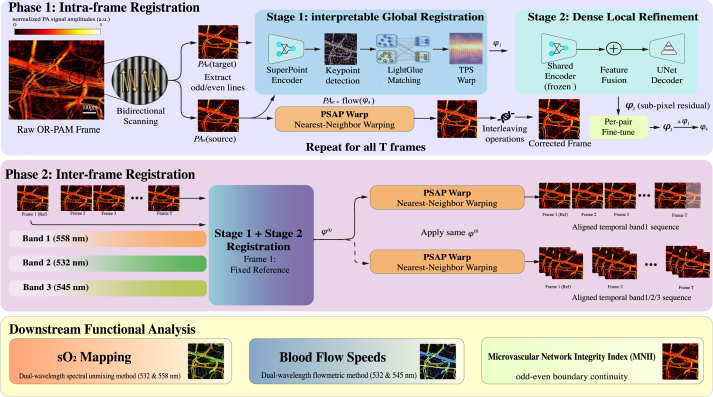
VITAL processing pipeline. Phase 1 (Intra-frame): odd/even sub-images are registered and interleaved into a corrected frame via nearest-neighbor warping to avoid interpolation-generated PA signal amplitudes. Phase 2 (Inter-frame): corrected frames are aligned to a reference; the band 1 deformation field is transferred to bands 2–3 for downstream sO${}_{2}$ mapping and $v_{\text{flow}}$ estimation.

The full pipeline runs in two phases (Fig. 5):

Phase 1 (Intra-frame): each frame is split into odd-line and even-line sub-images, registered with the two-stage model, and interleaved to reconstruct a corrected full-resolution frame.

Phase 2 (Inter-frame): the corrected full-resolution frames are aligned to the first frame. The same pretrained two-stage model is reused without retraining. Stage 1 estimates a global TPS field from keypoint correspondences between each frame $PA^{\left(t\right)}$ and the reference $PA^{\left(1\right)}$, while Stage 2 refines the remaining misalignment using a dense deformation field. Because inter-frame motion is primarily rigid or low-order, the resulting displacements are generally smaller than those in the intra-frame setting, enabling effective transfer of the pretrained model to inter-frame registration without additional sequence-specific retraining. The deformation is first estimated on band 1 (558 nm) and then applied to bands 2 (532 nm) and 3 (545 nm). This ensures consistent pixel correspondences across wavelengths for both sO${}_{2}$ mapping and $v_{\text{flow}}$ estimation, and is safer than independent per-band registration, which may introduce artificial spectral differences.

## 3 Experiments and results

### 3.1 Experimental setup

#### 3.1.1 Imaging system and dataset

To enable *in vivo* dynamic imaging of the mouse brain, we employ a stimulated Raman scattering (SRS)-based multi-wavelength OR-PAM system, as described in our previous work [4], [8], equipped with a voice-coil scanning stage for high-speed imaging. A schematic of the system is shown in Fig. 2a. A nanosecond pulsed laser (532-nm wavelength, VPFL-G-HE-30, Spectra-Physics) is used as a pump laser. Wavelength shifting is achieved via nonlinear propagation in optical fibers, where a 30-m polarization-maintaining single-mode fiber and a 100-m multimode fiber generate additional wavelengths at 545 nm and 558 nm through SRS. The three wavelengths are spectrally combined via optical components and coupled into the OR-PAM probe. Optical path length differences introduce controlled temporal delays between excitation pulses, enabling multi-wavelength photoacoustic signal generation.

To validate the performance of VITAL, *in vivo* oxygen challenge experiments were conducted in ICR mice, enabling downstream quantitative functional imaging analysis. Animals were anesthetized with isoflurane delivered via a mask. After anesthesia induction, the scalp and skull over the imaging region were carefully removed for imaging. During the experiment, the inhaled gas was switched from air to nitrogen gas (N${}_{2}$) for 125 s to induce hypoxia, followed by a return to air for recovery. Throughout the entire process, multi-wavelength OR-PAM imaging was continuously performed using a voice-coil scanning system operating at a bidirectional scanning speed of 30 Hz, generating a dynamic imaging dataset for analysis. At the end of the experiment, the mouse was euthanized according to standard protocols. All animal procedures were approved by the Animal Ethics Committee of the City University of Hong Kong.

The full dataset contains 681 odd–even image pairs collected across four independent acquisition sessions. Each odd–even image pair has a size of 250 × 500 pixels. For analysis, regions with stable imaging quality (150 × 300 pixels) were used for subsequent evaluation. After intra-frame registration and interleaving, they form a corrected 300 × 300 frame. For network processing, all inputs are zero-padded to 512 × 512. Across the pooled data from these four sessions, there are 227 paired multi-wavelength acquisitions, each contributing one odd–even pair at band 1, band 2, and band 3. The 227 band 1 pairs are used for model development and intra-frame evaluation, while the corresponding 454 pairs from band 2 and band 3 are warped using the deformation field estimated from band 1. For model development, these 227 pooled band 1 pairs were randomly divided into training and test sets with an 80/20 image-pair-level split, yielding 181 training pairs and 46 test pairs. We denote this held-out real OR-PAM test set as DI, which evaluates temporal generalization across held-out acquisition time points within the acquired dynamic imaging distribution. Inter-frame registration and downstream functional analysis are presented on one representative session with a complete 47-frame temporal sequence. Additional independent mouse ear-vessel validation experiments, denoted Dataset III (DIII; Mouse Ear I) and Dataset IV (DIV; Mouse Ear II), are provided in the Supplementary Material to assess external generalization across different vascular morphology and imaging conditions; these experiments are summarized in Supplementary Note S1 and Table S1, detailed in Supplementary Notes S4–S5 and Figures S2–S3, and included in the full benchmark summary in Table S4.

To further evaluate performance under controlled geometric deformation, we constructed a synthetic validation dataset, denoted as Dataset II (DII), from real OR-PAM vascular images. Synthetic moving images were generated from fixed images using a shared deformation across wavelength bands, preserving inter-band correspondence. The deformation consisted of random translation up to 28 pixels, random rotation up to 5°, no global scale change, and a smooth elastic random field generated on a 7 × 5 grid with elastic scale 1.0. The random seed was fixed at 443. For each synthetic pair, the moving-to-fixed displacement field and valid mask were saved together with the generated images. DII contains 48 synthetic odd–even pairs and is used only for validation. No model is trained on DII; the learning-based models trained on the DI training split are directly applied to DII, and the fine-tuning setting uses the same 300-step per-pair inference refinement as in DI. Representative DII visual examples are provided in Supplementary Note S6 and Figure S4.

#### 3.1.2 Implementation details

The framework is implemented in PyTorch. Stage 1 starts from pretrained SuperPoint and LightGlue weights, and the LightGlue matcher stays frozen throughout training. The SuperPoint encoder is frozen for the first 30 epochs while only the task-specific heads (the segmentation decoder and control-point regressor) are trained. It is then unfrozen for 70 more epochs with a learning rate reduced by 0.01× (100 epochs total, batch size 2, base learning rate 10^−4^). Training uses the 181 band 1 training pairs with standard augmentation including random flipping, 90° rotation, and intensity scaling. The loss is given by Eq. (10).

Stage 2 uses the same train–test split as Stage 1 and adopts the frozen Stage 1 encoder as its shared feature backbone. The UNet decoder is first trained for 500 epochs with the encoder frozen. The encoder is then unfrozen, the learning rate is lowered by 0.01×, and training continues with validation-based early stopping. After that, the hyperparameters are refined through five rounds of automated search, for about 2500 aggregate training epochs. Automatic mixed-precision (AMP) training and TorchScript just-in-time (JIT) compilation are enabled for faster inference. At test time, Stage 2 produces an initial residual deformation field in a single forward pass and then refines it for 300 steps per pair (Section 2.5). The band 1 deformation field is finally applied to bands 2 and 3 using nearest-neighbor warping. All experiments use an NVIDIA RTX 4090 GPU with 512 × 512 single-channel inputs.

VoxelMorph and TransMorph were trained from scratch rather than initialized from pretrained OR-PAM registration models. To ensure a comparable training and evaluation protocol, both baselines used the same 181 band 1 training pairs, the same 46 held-out band 1 test pairs, the same 512 × 512 input size, the same data augmentation, the same optimizer type, the same learning-rate schedule, the same maximum training epochs, the same validation-based early stopping strategy, and the same metric implementation as VITAL Stage 2. Following the standard unsupervised objectives used in the original VoxelMorph and TransMorph frameworks, these baselines were trained with an NCC-based image similarity loss and deformation-field smoothness regularization. Thus, the comparison uses the same data split, training budget, and evaluation pipeline, while preserving the original loss design of each baseline method.

#### 3.1.3 Evaluation metrics

Registration quality is evaluated with six metrics. Spatial alignment is measured by MSE, NCC, SSIM, and peak signal-to-noise ratio (PSNR), all computed inside a method-independent binary vessel ROI generated from the fixed/target image using Otsu thresholding and identical post-processing. This ROI is fixed for each image pair and shared by VITAL and all baseline methods. PFr assesses preservation of measured PA signal distributions during registration by quantifying interpolation-induced alterations in PA signal amplitudes, including generation of interpolated values, loss of source-value bins, and redistribution of value frequencies. The use of both metric groups reflects that spatial alignment alone is insufficient for quantitative functional OR-PAM. Registration-quality metrics evaluate geometric correspondence, whereas PFr evaluates preservation of measured PA signal distributions. High spatial-registration scores support accurate anatomical correspondence, while high PFr indicates reduced alteration of measured PA signal distributions during registration. Together, these complementary metrics provide a more comprehensive assessment of PA registration reliability.

Standard spatial metrics quantify structural alignment but do not evaluate preservation of measured PA signal distributions during registration. A method may achieve high NCC or SSIM scores while still altering PA signal amplitudes through interpolation. To quantify this distribution fidelity, PFr is defined as a histogram-intersection metric for measured PA signal-amplitude distribution preservation during registration. Prior to histogram construction, PA signal amplitudes and derived sO${}_{2}$ values are uniformly quantized to an integer range of [0, 10000], serving as discrete bins for value-frequency comparison. Let $R_{\text{src}}$ and $R_{\text{tgt}}$ denote the regions of interest in the source and target coordinate frames, respectively. The source histogram $h_{\text{src}}^{\;R_{\text{src}}}\left(v\right)$ counts pixels falling into the vth quantized value bin within $R_{\text{src}}$, while the registered histogram $h_{\text{reg}}^{\;R_{\text{tgt}}}\left(v\right)$ counts pixels within $R_{\text{tgt}}$ (background zeros excluded in both cases). In this work, $R_{\text{tgt}}$ is the method-independent Otsu-derived vessel ROI generated from the fixed/target image, and $R_{\text{src}}$ is generated from the source image using the same Otsu-thresholding and post-processing procedure. This ROI construction is independent of the registration method and is applied identically to VITAL and all baselines. PFr is defined as

(28) $$\text{PFr}=\frac{\underset{v}{\sum }\min\;\left(\overset{\;R_{\text{tgt}}}{\underset{\text{reg}}{h}}\left(v\right),\;h_{\text{src}}^{\;R_{\text{src}}}\left(v\right)\right)}{\underset{v}{\sum }h_{\text{src}}^{\;R_{\text{src}}}\left(v\right)}\times 100\text{\%}$$

PFr is affected by two aspects of registration behavior. First, if geometric alignment is inaccurate, vessel structures in the registered image deviate from $R_{\text{tgt}}$, substantially reducing the number of vessel pixels within the target region and causing a systematic mismatch between $h_{\text{reg}}^{\;R_{\text{tgt}}}$ and $h_{\text{src}}^{\;R_{\text{src}}}$. Second, even when geometric alignment is accurate, if the registration method introduces interpolation-generated PA amplitudes or redistributes source amplitudes, the resulting distribution alteration likewise reduces PFr. Importantly, PFr should not be interpreted as a standalone measure of spatial registration accuracy or quantitative correctness. Rather, it evaluates preservation of the measured PA signal-amplitude distribution during registration: a higher PFr indicates smaller alteration of source PA signal distributions across the corresponding source and target vessel ROIs, with fewer interpolation-generated values, fewer lost source-value bins, and less redistribution of value frequencies. Therefore, PFr complements, rather than replaces, spatial metrics such as MSE, NCC, SSIM, and PSNR. In this work, registration performance is interpreted jointly from spatial alignment metrics, PFr, and downstream functional analyses. The corresponding distribution-level behavior is further visualized in Section 3.5.3 using value-frequency scatter plots.

Vascular continuity is further evaluated using the Microvascular Network Integrity Index (MNII), which quantifies continuity across odd–even scan lines. For computation, a 1-pixel interleaved composite image is constructed, in which the odd-line image and the registered even-line image occupy alternating columns. These alternating columns correspond directly to the original odd and even scan lines in image form. The composite is then separated back into odd-line and even-line channels. Let O denote the odd-line image, E denote the registered even-line image, and Ω denote the vessel-support region. The odd–even discrepancy is defined as

(29) $$D_{\text{oe}}=\frac{1}{\left|\Omega \right|}\underset{\left(i,j\right)\in \Omega }{\sum }\frac{\left|O_{i,j}-E_{i,j}\right|}{k_{\text{ref}}},$$

where $k_{\text{ref}}$ is a reference normalization scale reflecting the characteristic magnitude of inter-line signal variation in vessel regions. The final MNII is obtained through a monotonic decreasing mapping:

(30) $$\text{MNII}=N\left(D_{\text{oe}}\right),$$

where N(⋅) converts the normalized discrepancy into a bounded continuity score. Under this definition, lower odd–even discrepancy yields higher MNII, indicating better continuity of vascular structures across interleaved scan lines.

#### 3.1.4 Baseline methods

We compare VITAL with six representative registration baselines: SIFT [16], Optical Flow [17], Demons [18], SyN (Advanced Normalization Tools, ANTs) [19], VoxelMorph [20], and TransMorph [21]. The original baseline outputs use interpolation-based final resampling, as commonly used for scalar intensity-image registration. For the two learning-based baselines, we additionally report nearest-neighbor (NN) final-resampling controls, denoted as VoxelMorph (NN) and TransMorph (NN), in which the deformation field predicted by the baseline model is applied to the raw source image using nearest-neighbor warping. These controls reuse the same predicted deformation fields and replace only the final resampling step, allowing deformation estimation and signal-value resampling fidelity to be examined separately.

### 3.2 Intra-frame registration comparison

Table 1 reports the main quantitative comparison of all methods on the DI real OR-PAM test set and the DII synthetic deformation validation set, evaluated within the masked ROI.

VITAL consistently achieves the best performance across all reported metrics in Table 1. On DI, direct VITAL achieves MSE = 0.0144±0.0139, NCC = 0.716±0.139, SSIM = 0.727±0.074, PSNR = 20.58±4.48 dB, and PFr = 97.33±0.86%. After fine-tuning, VITAL further improves to MSE = 0.0072±0.0054, NCC = 0.821±0.046, SSIM = 0.804±0.027, PSNR = 22.63±3.25 dB, and PFr = 97.83±0.82%. On DII, direct VITAL achieves MSE = 0.0066±0.0014, NCC = 0.915±0.018, SSIM = 0.826±0.024, PSNR = 21.90±0.92 dB, and PFr = 98.44±0.67%. After fine-tuning, VITAL reaches MSE = 0.0060±0.0012, NCC = 0.923±0.015, SSIM = 0.841±0.021, PSNR = 22.34±0.88 dB, and PFr = 98.33±0.66%. These results show consistent performance in both cross-time-point real-image validation and controlled synthetic deformation.

**Table 1:** Main quantitative comparison on the DI real held-out test set and the DII synthetic deformation validation set. Direct apply denotes applying each method without the additional 300-step learning-based per-pair fine-tuning: trained learning-based models are evaluated with fixed weights, while traditional pairwise baselines are reported using their standard registration outputs. Fine-tuning denotes 300-step per-pair optimization initialized from the direct-apply output for learning-based methods. Values are reported as mean ± std where applicable; PFr is computed within the ROI and is not reported for unregistered inputs. For TransMorph and VoxelMorph, BL denotes the original bilinear final-resampling output, whereas NN denotes the nearest-neighbor final-resampling control applied to the same predicted deformation field. **Bold**: best.

Dataset	Method	Direct apply					Fine-tuning				
		MSE ↓	NCC ↑	SSIM ↑	PSNR ↑	PFr (%) ↑	MSE ↓	NCC ↑	SSIM ↑	PSNR ↑	PFr (%) ↑
DI	Unregistered	0.0660±0.0423	0.178±0.140	0.129±0.067	12.84±3.12	–	–				
DI	SIFT	0.0565±0.0310	0.277±0.163	0.222±0.053	13.18±2.56	13.16±1.21	–				
DI	Optical Flow	0.0764±0.0431	0.110±0.109	0.088±0.052	11.91±2.63	12.14±0.79	–				
DI	Demons	0.0457±0.0208	0.435±0.180	0.422±0.082	13.93±2.28	12.88±0.95	–				
DI	SyN	0.0240±0.0127	0.649±0.154	0.669±0.059	16.80±2.34	14.59±0.59	–				
DI	TransMorph (BL)	0.0271±0.0164	0.683±0.227	0.663±0.138	16.42±2.60	14.50±0.61	0.0261±0.0159	0.694±0.227	0.696±0.141	16.62±2.65	21.45±2.32
DI	TransMorph (NN)	0.0249±0.0174	0.673±0.222	0.680±0.145	17.00±2.88	20.75±2.48	0.0243±0.0169	0.685±0.221	0.696±0.143	17.09±2.85	20.83±2.46
DI	VoxelMorph (BL)	0.0263±0.0174	0.677±0.215	0.626±0.156	16.58±2.54	14.65±0.54	0.0247±0.0173	0.715±0.215	0.683±0.167	16.99±2.77	21.54±2.57
DI	VoxelMorph (NN)	0.0347±0.0219	0.498±0.196	0.469±0.114	15.51±2.92	19.33±2.39	0.0335±0.0210	0.512±0.190	0.483±0.115	15.62±2.82	19.51±2.41
DI	VITAL	**0.0144**±**0.0139**	**0.716**±**0.139**	**0.727**±**0.074**	**20.58**±**4.48**	**97.33**±**0.86**	**0.0072**±**0.0054**	**0.821**±**0.046**	**0.804**±**0.027**	**22.63**±**3.25**	**97.83**±**0.82**

DII	Unregistered	0.0838±0.0111	−0.104±0.100	0.031±0.030	10.81±0.61	–	–				
DII	SIFT	0.0212±0.0044	0.724±0.057	0.500±0.085	16.83±0.92	95.95±1.78	–				
DII	Optical Flow	0.0749±0.0081	0.021±0.068	0.045±0.016	11.28±0.48	58.21±4.61	–				
DII	Demons	0.0572±0.0147	0.232±0.180	0.233±0.114	12.60±1.31	72.64±5.36	–				
DII	SyN	0.0831±0.0111	−0.085±0.100	0.035±0.027	10.84±0.61	64.27±4.33	–				
DII	TransMorph (BL)	0.0615±0.0124	0.202±0.145	0.177±0.062	12.21±0.96	75.57±4.21	0.0379±0.0089	0.510±0.113	0.466±0.075	14.36±1.16	88.41±2.60
DII	TransMorph (NN)	0.0619±0.0120	0.196±0.138	0.166±0.060	12.17±0.90	73.10±5.36	0.0379±0.0089	0.509±0.109	0.456±0.079	14.35±1.15	85.57±4.86
DII	VoxelMorph (BL)	0.0613±0.0172	0.209±0.206	0.185±0.103	12.34±1.46	76.50±5.15	0.0441±0.0172	0.426±0.222	0.351±0.176	14.09±2.55	86.39±4.52
DII	VoxelMorph (NN)	0.0612±0.0163	0.201±0.197	0.174±0.100	12.32±1.36	73.95±6.51	0.0446±0.0173	0.416±0.219	0.341±0.184	14.05±2.59	83.36±7.96
DII	VITAL	**0.0066**±**0.0014**	**0.915**±**0.018**	**0.826**±**0.024**	**21.90**±**0.92**	**98.44**±**0.67**	**0.0060**±**0.0012**	**0.923**±**0.015**	**0.841**±**0.021**	**22.34**±**0.88**	**98.33**±**0.66**

On DI, among the traditional methods, SyN achieves highest NCC (0.649±0.154), but it still trails VITAL. SIFT improves NCC and slightly reduces MSE, but the improvement remains modest compared with VITAL. Optical Flow performs worst on NCC (0.110±0.109) and degrades all four spatial metrics below the unregistered baseline, indicating that signal-consistency-based registration is counterproductive under strong PA amplitude inconsistency. Among the learning-based baselines, TransMorph reaches NCC = 0.683±0.227 with MSE = 0.0271±0.0164, and VoxelMorph achieves NCC = 0.677±0.215 with MSE = 0.0263±0.0174. The NN-resampling controls further separate deformation estimation from the final resampling strategy. In contrast, VITAL combines high spatial accuracy with high measured PA signal-amplitude distribution preservation as quantified by PFr, while maintaining low variance across metrics (e.g. NCC std = 0.046). Runtime is 0.12 s per 512 × 512 pair in direct-apply mode and 3.33 s per pair in fine-tuning mode on an NVIDIA RTX 4090 GPU. Thus, the key trade-off is not speed but whether the method preserves the measured PA signal-amplitude distribution during quantitative analysis.

**Fig. 6:**
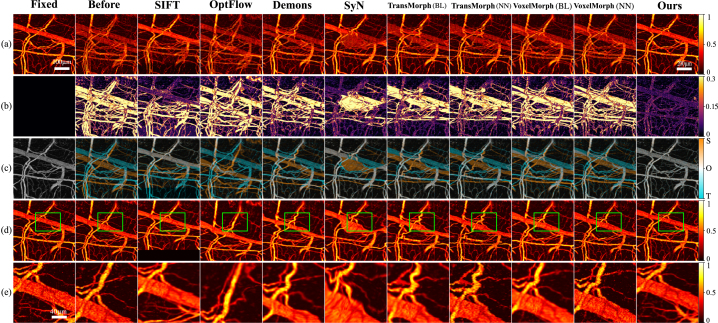
Visual comparison of intra-frame registration performance. (a) Full field-of-view images of the target image (odd lines only), the unregistered odd–even interleaved image, and the registered results obtained by different methods. (b) and (c) show absolute difference maps and color overlays with respect to the target odd-line image, including the self-baseline and the corresponding even-line images from the unregistered and registered results. Lower residual intensity and stronger structural overlap indicate better alignment. (d) From left to right: the target odd-line image, the source even-line image before registration, and the registered even-line images produced by different methods. (e) Magnified views of the region highlighted by the green box in (d), showing that VITAL achieves the most continuous vascular structures with minimal boundary artifacts. From top to bottom, the colorbars represent normalized PA signal intensity (0–1) for (a), (d), and (e); absolute PA signal-intensity difference (0–0.3) for (b); and source–target overlay categories (S: source, O: overlap, T: target) for (c). In the method labels, BL denotes the original bilinear final-resampling output, whereas NN denotes the nearest-neighbor final-resampling control applied to the same predicted deformation field.

The PFr results further separate deformation accuracy from final resampling behavior. On DI, the original interpolation-based baselines remain between 12.14% and 14.65% PFr in direct apply. The additional TransMorph (NN) and VoxelMorph (NN) controls increase PFr relative to their bilinear counterparts but remain far below VITAL, indicating that nearest-neighbor final resampling alone is insufficient without accurate deformation estimation. On DII, SIFT also preserves a high PFr under controlled synthetic deformation (95.95% ± 1.78%), but its spatial accuracy remains lower than VITAL (MSE 0.0212 vs. 0.0066; NCC 0.724 vs. 0.915 in direct apply). This reinforces that PFr should be interpreted together with spatial metrics rather than as an isolated score. In OR-PAM, accurate registration is insufficient if the transformation alters signal amplitudes used for quantitative analysis. By employing nearest-neighbor warping, VITAL keeps registered PA signal amplitudes traceable to existing uint16 source PA amplitudes and substantially improves measured PA signal-amplitude distribution preservation compared with interpolation-based methods. As shown in Fig. 6, this keeps downstream measurements tied to acquired source amplitudes rather than synthetic intermediate amplitudes.

### 3.3 Intra-frame registration ablation study

All ablation experiments are evaluated on the DI real held-out test set. Table 2 summarizes three ablation groups: the coarse-to-fine architecture, nearest-neighbor warping, and per-pair fine-tuning.

**Table 2:** Ablation study on framework components on the DI real held-out test set (masked ROI). Values are reported as mean ± std. Each row removes or modifies one design component relative to the full model. **Bold**: best; underline: second best.

	Configuration	MSE ↓	NCC ↑	SSIM ↑	PSNR ↑	PFr (%) ↑
*(I) Architecture: Coarse-to-fine design*						
(a)	S2 only (w/o Stage 1)	0.0173±0.0138	0.736±0.065	0.659±0.107	19.29±4.05	94.03±3.76
(b)	S1 only (w/o Stage 2)	0.0200±0.0162	0.614±0.122	0.600±0.054	18.42±3.55	95.05±1.30

*(II) Nearest-neighbor warping*						
(c)	Bilinear interpolation	0.0072±0.0057	**0.877**±**0.034**	**0.845**±**0.028**	**22.70**±**3.34**	16.74±0.82

*(III) Per-pair fine-tuning*						
(d)	w/o fine-tuning (direct apply)	0.0144±0.0139	0.716±0.139	0.727±0.074	20.58±4.48	97.33 ± 0.86

	**VITAL (Full)**	**0.0072**±**0.0054**	0.821 ± 0.046	0.804 ± 0.027	22.63 ± 3.25	**97.83**±**0.82**

#### 3.3.1 Architecture ablation

The ablation study clarifies the division of roles within the architecture. Stage 2 alone (row a in Table 2) achieves higher spatial accuracy than Stage 1 alone (row b in Table 2), but still falls short of the full model across all spatial metrics (e.g., NCC 0.736 vs. 0.821 and PSNR 19.29 vs. 22.63 dB). Both single-stage variants achieve lower PFr than the full model (94.03% for S2 only, 95.05% for S1 only vs. 97.83%), suggesting that incomplete geometric correction can also reduce preservation of the measured PA signal-amplitude distribution. However, Stage 1 alone lacks the local refinement necessary for precise alignment. The full model performs best because Stage 1 provides global correction while Stage 2 refines the residual error. Neither stage is redundant, as each addresses a distinct aspect of the problem.

#### 3.3.2 Nearest-neighbor warping ablation

The result in row c in Table 2 highlights a key trade-off in VITAL. Bilinear interpolation improves NCC from 0.821 to 0.877 and SSIM from 0.804 to 0.845, while PSNR changes only slightly from 22.63 to 22.70 dB and MSE remains essentially unchanged at 0.0072. Equivalently, compared with bilinear interpolation, nearest-neighbor warping decreases NCC by 0.056 (6.39%), SSIM by 0.041 (4.85%), and PSNR by 0.07 dB (0.31%). In return, PFr increases by 81.09 percentage points, from 16.74% to 97.83%. Thus, the spatial cost of nearest-neighbor final warping is measurable but limited, whereas PFr indicates stronger preservation of the measured PA signal-amplitude distribution. The higher NCC and SSIM obtained with bilinear interpolation mainly reflect visually smoother vessel boundaries and improved image-similarity scores. However, these gains are accompanied by a marked decrease in PFr, indicating stronger alteration of measured PA signal-amplitude distribution through interpolation-generated amplitudes and value-frequency redistribution. This effect is particularly important for functional OR-PAM, because sO${}_{2}$ and $v_{\text{flow}}$ are computed from PA signal amplitudes from multiple wavelengths. Independently smoothing registered PA images across different wavelength bands therefore does not simply produce smoother functional maps, but can also affect sO${}_{2}$ and $v_{\text{flow}}$ estimation. We therefore adopt nearest-neighbor warping for quantitative OR-PAM, prioritizing preservation of measured PA signal amplitudes over visual smoothness.

#### 3.3.3 Per-pair fine-tuning ablation

Without per-pair fine-tuning (row d), the model remains competitive, but all four alignment metrics degrade: MSE increases to 0.0144, and NCC and SSIM drop to 0.716 and 0.727. PFr also slightly decreases from 97.83% to 97.33%, indicating slightly lower preservation of the measured PA signal-amplitude distribution after registration. Runtime is reduced from 3.33 s per pair in fine-tuning mode to 0.12 s per pair in direct-apply mode, revealing a clear trade-off: fine-tuning improves alignment and PFr, whereas direct application is faster. We therefore adopt the fine-tuned setting as the main configuration because quantitative analysis relies on both accurate correspondence and preservation of the measured PA signal-amplitude distribution, while the direct-apply mode remains practical under throughput constraints.

### 3.4 Vascular continuity analysis

Table 3 reports MNII for all methods.

**Fig. 7:**
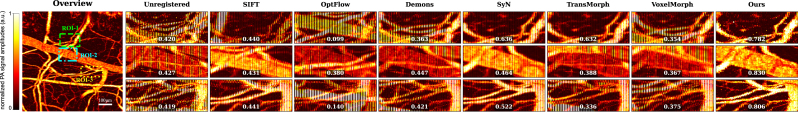
Vascular continuity comparison across three ROIs. Left: overview with ROI locations—ROI-1 (green), ROI-2 (cyan), ROI-3 (yellow). Right: zoomed-in comparison across methods for each ROI with per-ROI MNII values. VITAL achieves the highest continuity across all three ROIs.

VITAL achieves an MNII of 0.876, outperforming all baselines and exceeding the unregistered case (0.587) by 49.2%. The closest competitors are VoxelMorph (0.680) and TransMorph (0.657). Optical Flow falls below the unregistered case at 0.417, indicating that intensity-driven registration can degrade continuity under strong PA amplitude inconsistency. Fig. 7 shows three representative ROIs: ROI-1, ROI-2, and ROI-3. VITAL produces the most continuous vascular structures in all cases. This consistency is critical, as continuity errors directly impair vessel-based measurements, affecting both network topology and downstream functional parameters.

**Table 3:** Microvascular Network Integrity Index (MNII) comparison. **Bold**: best.

Method	MNII ↑
Unregistered	0.587
SIFT	0.484
Optical Flow	0.417
Demons	0.528
SyN (ANTs)	0.601
TransMorph	0.657
VoxelMorph	0.680
**VITAL (Ours)**	**0.876**

### 3.5 Downstream functional imaging applications

Quantitative functional imaging requires both accurate spatial alignment and preservation of the measured PA signal-amplitude distribution after registration. Accordingly, we analyze a representative 47-frame sequence using two sensitive tasks – sO${}_{2}$ mapping and $v_{\text{flow}}$ estimation – to evaluate not only visual quality but also to assess whether the registered sequence remains suitable for downstream functional analysis.

After intra-frame correction, all frames are registered to a common reference. Without additional sequence-specific retraining, the pretrained VITAL model achieves average values of NCC = 0.892±0.009, SSIM = 0.599±0.022, PSNR = 20.80±0.40 dB, and MSE = 0.0083±0.0008 for inter-frame alignment. These results indicate that frame-to-frame correspondence remains sufficiently stable in this representative sequence to support temporal sO${}_{2}$ mapping and $v_{\text{flow}}$ estimation. Additional inter-frame registration results and the functional-analysis scope are provided in Supplementary Note S7 and Table S3.

#### 3.5.1 sO${}_{2}$ mapping

Relative sO${}_{2}$ is estimated from dual-wavelength spectral unmixing method based on PA signals at 558 nm and 532 nm [4], [7], [8]. Because this method combines multi-wavelength signals pixel by pixel, interpolation-generated PA amplitudes in individual bands can alter the spectral relationships before unmixing and propagate nonlinearly into sO${}_{2}$ estimation. Similarly, $v_{\text{flow}}$ estimation depends on temporal PA signal changes, so interpolation-induced redistribution can affect the temporal relationships used for flow analysis. For this reason, VITAL applies the same deformation field to the wavelength bands using nearest-neighbor final resampling, keeping the functional calculation tied to measured source PA amplitudes. For downstream functional analysis, the soft vessel mask predicted by the segmentation decoder (Section 2.2.3) is used to restrict sO${}_{2}$ and $v_{\text{flow}}$ analysis to vascular regions. This functional mask is separate from the Otsu-derived method-independent ROI used for registration metric evaluation. The resulting sO${}_{2}$ values reliably capture temporal changes and differences, yet remain sensitive to registration quality due to their pixel-wise dependence. Absolute calibration would require additional phantom Refs. [8].

**Fig. 8:**
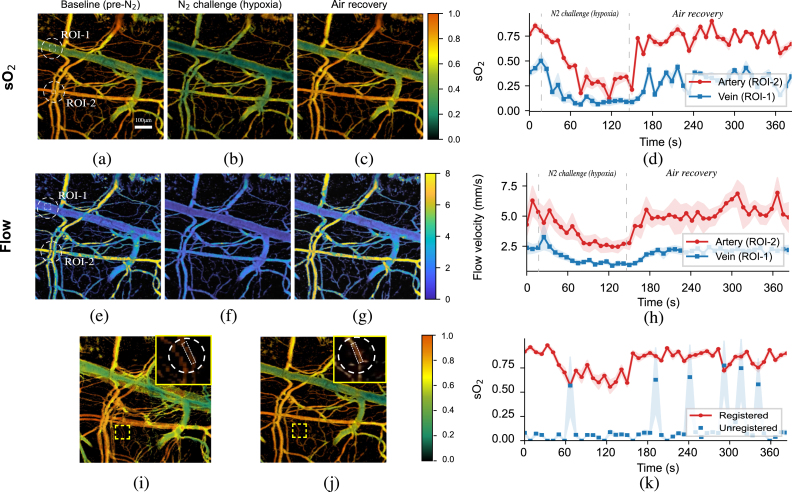
Functional measurements before and after VITAL-based registration. (a)–(c) and (e)–(g) show the temporal evolution of sO${}_{2}$ and $v_{\text{flow}}$ in ROI-1 (vein) and ROI-2 (artery) during the oxygen challenge experiment. (d) and (h) present the full temporal profiles of sO${}_{2}$ and $v_{\text{flow}}$. (i)–(k) illustrate the effect of registration on microvascular sO${}_{2}$ analysis: (i) and (j) show sO${}_{2}$ before and after registration in a representative ROI (vessels located within the dashed box), and (k) shows temporal sO${}_{2}$ change process for the registered (red) and unregistered (blue) data, with shaded error bands.

Fig. 8a–c shows sO${}_{2}$ mapping for three physiological states. After registration, the alternating stripe artifacts at odd–even boundaries are eliminated. During oxygen challenge, the arterial ROI shows a clear decrease in sO${}_{2}$ under N${}_{2}$ followed by recovery under air, whereas the venous ROI exhibits more moderate changes (Fig. 8d), consistent with expected physiology.

Without accurate registration, such microvascular dynamics may be obscured even when the underlying physiology remains intact. Fig. 8i–k illustrates the effect of registration on microvascular sO${}_{2}$ analysis. Before registration (Fig. 8i), the sO${}_{2}$ mapping shows obvious stripe artifacts along odd–even boundaries. After registration (Fig. 8j), microvascular misalignment is substantially reduced. The temporal traces in Fig. 8k show the same trend: the registered curve captures the decrease under N${}_{2}$ and subsequent recovery under air, whereas the unregistered curve fluctuates irregularly because of misalignment artifacts. This comparison demonstrates how registration errors can lead to misinterpretation of physiological signals if uncorrected.

#### 3.5.2 $v_{\text{flow}}$ Estimation

After inter-frame registration, all temporal frames share a common coordinate system, enabling temporally consistent $v_{\text{flow}}$ analysis. Relative $v_{\text{flow}}$ is estimated using a previously reported dual-wavelength (545 nm and 532 nm) flowmetric method [8]. Without registration, frame-to-frame misalignment introduces spatial artifacts that distort functional results and obscure true temporal dynamics.

Fig. 8e–g shows the resulting $v_{\text{flow}}$ under three physiological states. After VITAL registration, the $v_{\text{flow}}$ maps become spatially coherent, remain confined to vessels, and exhibit stronger temporal variation in arteries than in venules. Supplementary Video S1 shows the dynamic change of sO${}_{2}$ and $v_{\text{flow}}$ during the entire oxygen challenge experiment. In this representative sequence, VITAL supports $v_{\text{flow}}$ estimation by restoring frame-to-frame correspondence and maintaining the link between measured changes and vascular anatomy.

#### 3.5.3 Distribution fidelity analysis of PA signal amplitudes and sO${}_{2}$ mapping

**Fig. 9:**
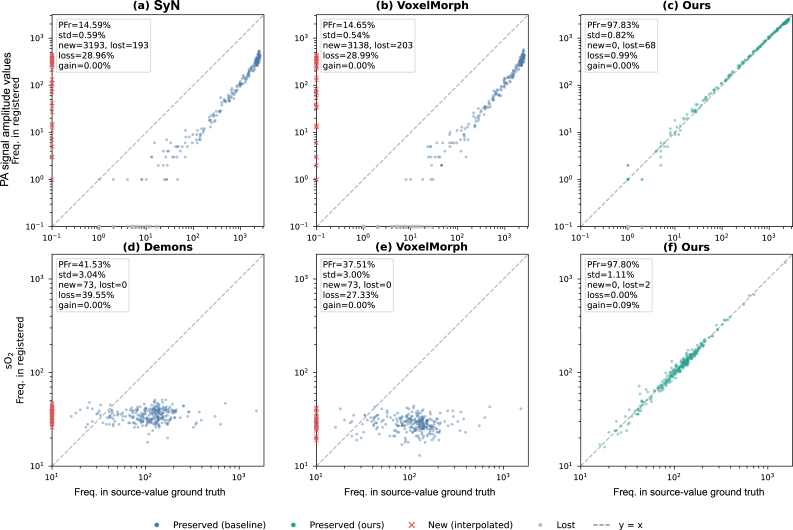
Distribution fidelity analysis of PA signal amplitudes (top row) and derived sO${}_{2}$ (bottom row). Each subplot shows a value-frequency scatter plot on log–log axes, where the horizontal axis represents the frequency of each quantized value bin in the source image and the vertical axis represents the frequency of the same bin in the registered image. Three point categories are shown: preserved (circles), quantized value bins present in both images, with proximity to the diagonal (y=x) indicating faithful frequency preservation; new (crosses, plotted on the vertical axis at zero source frequency), quantized value bins introduced after registration; and lost (squares, plotted on the horizontal axis at zero registered frequency), source bins that disappear after registration. Inset statistics report PFr, the numbers of new and lost bins, and overall pixel-count loss and gain rates. VITAL produces the tightest diagonal clustering, consistent with its superior PFr in Table 1, Table 4.

To evaluate how preservation of the measured PA signal-amplitude distribution propagates through the functional pipeline, Fig. 9 compares value-frequency distributions at two levels: PA signal amplitudes and the derived sO${}_{2}$ maps. To construct the scatter plots, the source and registered images are first quantized to an integer range of [0, 10000] and converted into value-frequency histograms. Each point corresponds to a quantized value bin, with the horizontal and vertical axes representing its frequency in the source and registered images, respectively. Points near the diagonal y=x indicate better preservation of the original value-frequency distribution. This analysis complements spatial metrics by evaluating not only alignment but also whether the underlying measurement distribution is preserved after registration.

In Fig. 9, each point can be classified into one of three categories. Preserved points (circles) are quantized value bins present in both the source and registered images, with proximity to the diagonal indicating better preservation. New points (crosses, plotted on the vertical axis) represent quantized value bins absent from the source image but introduced after registration, typically due to interpolation; their source frequency is zero. Lost points (squares, plotted on the horizontal axis) represent quantized value bins present in the source image but missing after warping; their registered frequency is zero. We further report two summary statistics: loss, defined as the fraction of source pixel counts that vanish after warping, and gain, defined as the fraction of additional pixel counts introduced relative to the source total.

According to Fig. 9, baseline methods generate considerable new bins and exhibit broad off-diagonal dispersion among preserved bins, indicating that interpolation introduces intermediate amplitudes in such conventional methods. Many preserved points fall below the diagonal, manifesting that bins frequent in the source image become less frequent after registration due to redistribution into interpolated amplitudes. In contrast, VITAL produces minimal new bins and maintains tight clustering around the diagonal, achieving PFr = 97.83% (band 1) and 97.80% (sO${}_{2}$), higher than the baselines. This pattern underscores that preserving the measured PA signal-amplitude distribution is critical not only for the raw image, but also for the derived functional readouts.

Table 4 presents the comparison at the sO${}_{2}$-mapping level. VITAL achieves the best performance across all five metrics: NCC = 0.796±0.038, SSIM = 0.638±0.047, PSNR = 15.90±2.15 dB, MSE = 0.0288±0.0126, and sO${}_{2}$-level PFr = 97.80%. TransMorph is the closest baseline on the spatial metrics, with NCC = 0.749±0.078, but its sO${}_{2}$ PFr is only 37.16%, which indicates substantial distortion propagated into the derived maps. In fact, all baselines remain below 42% PFr at the sO${}_{2}$ level, with Demons highest at 41.53%. Notably, Optical Flow and SyN reduce NCC below the unregistered case, indicating that intensity-driven registration may introduce incorrect deformations and degrade downstream functional analysis. These results reveal a measurable gap between spatial quality and functional reliability. In the representative sequence, VITAL supports sO${}_{2}$ estimation after registration rather than merely improving visual appearance.

**Table 4:** Quantitative comparison of sO${}_{2}$ mapping quality across registration methods. Values are reported as mean ± std. **Bold**: best.

Method	MSE ↓	NCC ↑	SSIM ↑	PSNR ↑	PFr (%) ↑
Unregistered	0.0688±0.0313	0.510±0.051	0.433±0.023	12.26±2.58	–

SIFT	0.0647±0.0230	0.533±0.072	0.457±0.026	12.30±2.10	40.75±3.37
Optical Flow	0.0737±0.0340	0.478±0.061	0.426±0.023	12.01±2.71	39.95±3.02
Demons	0.0519±0.0220	0.612±0.051	0.514±0.043	13.40±2.39	41.53±3.04
SyN (ANTs)	0.0702±0.0300	0.488±0.052	0.433±0.022	12.08±2.38	37.38±3.13
TransMorph	0.0348±0.0173	0.749±0.078	0.612±0.074	15.26±2.55	37.16±3.01
VoxelMorph	0.0434±0.0205	0.680±0.068	0.547±0.056	14.26±2.53	37.51±3.00
**VITAL (Ours)**	**0.0288**±**0.0126**	**0.796**±**0.038**	**0.638**±**0.047**	**15.90**±**2.15**	**97.80**±**1.11**

## 4 Discussion

In this work, the framework is designed to satisfy two practical requirements: correcting spatial misalignment caused by intra-frame and inter-frame artifacts while preserving the measured PA signal-amplitude distribution for downstream quantification. VITAL achieves this through a stage-wise design. Stage 1 resolves large distortions (5–20 pixels) using explicit vascular correspondences that remain interpretable under amplitude variation. Stage 2 then operates in a regime (0.6–1.4 pixels) where smoothness and Jacobian constraints ensure stable refinement. Nearest-neighbor final resampling and PFr then address the complementary requirement of measured PA signal-amplitude distribution preservation: the registered PA signal amplitudes remain traceable to acquired source amplitudes, and PFr assesses preservation of the measured PA signal-amplitude distribution during registration. This distinction is important because spatial correctness and measured PA signal-amplitude distribution preservation are both required for quantitative PA reliability, which directly affects downstream functional calculations. Averaging-type interpolation can make registered PA amplitudes appear smoother, but sO${}_{2}$ and $v_{\text{flow}}$ are functions of multi-wavelength or temporal PA measurements; smoothing each PA image independently therefore does not guarantee a smoother or more reliable functional map and may instead perturb the spectral or temporal relationships used for functional estimation. High spatial metrics indicate improved geometric correspondence, whereas high PFr together with nearest-neighbor resampling indicates reduced alteration of measured PA signal distributions during registration. Together, these complementary metric groups suggest that the registered sequence maintains both stable spatial correspondence and reduced interpolation-induced signal distortion, which is important for downstream sO${}_{2}$ and $v_{\text{flow}}$ analysis.

Bidirectional voice-coil scanning was adopted in this study because it provides a practical balance among imaging speed, field of view (FOV), detection sensitivity, spatial resolution, and multi-wavelength imaging capability for quantitative functional OR-PAM. While alternative high-speed scanning architectures provide different trade-offs among imaging speed, FOV, sensitivity, and system complexity, quantitative functional imaging has additional requirements beyond imaging speed alone. Reliable estimation of physiological parameters such as sO${}_{2}$ and $v_{\text{flow}}$ requires sufficient photoacoustic SNR across multiple excitation wavelengths, making detection sensitivity an important practical consideration in multi-wavelength functional imaging. Under these constraints, voice-coil scanners remain attractive because they allow flexible adjustment of imaging speed and FOV while maintaining high PA signal sensitivity and lateral resolution. The spatial misalignment observed in voice-coil-based OR-PAM originates primarily from forward–backward trajectory asymmetry, mechanical hysteresis, temporal sampling asymmetry, scanner vibration, and long-term actuator drift. Although hardware optimization, calibration, and improved control strategies can alleviate these effects, residual mismatch often remains in practical *in vivo* imaging because of physiological motion, tissue deformation, and environmental variability. Consequently, hardware optimization and computational correction should be viewed as complementary rather than mutually exclusive solutions.

NCC and SSIM should therefore be interpreted as spatial similarity indicators, and higher values should be considered in the context of the registration task and downstream quantitative analysis. This is especially important for both intra-frame and inter-frame registration in *in vivo* dynamic OR-PAM. Consecutive frames, and even odd/even sub-images acquired within a dynamic scan, are not expected to be identical in PA signal amplitude. Red blood cell flow, local hemoglobin-concentration fluctuation, oxygenation changes, and physiological motion can all produce real PA signal variation. A method that drives NCC or SSIM excessively high may therefore improve visual similarity by over-smoothing, over-matching, or suppressing real dynamic changes, rather than improving functional quantification. PFr addresses a complementary question: how much the PA signal-amplitude distribution changes during registration. This helps evaluate whether spatial misalignment has moved a large number of nonvascular or mismatched PA amplitudes into the vessel ROI, and whether interpolation has generated many interpolation-generated PA amplitudes that were not present in the acquired source data. For this reason, PFr is used together with spatial metrics, not as their replacement, to assess whether registration is both geometrically consistent and quantitatively suitable for downstream functional analysis.

SuperPoint pretrained on natural images [25] can be adapted to OR-PAM vascular data because the relevant low-level structures are partly shared. SuperPoint learns generic corners and edges that correspond to vascular structures such as bifurcations, crossings, and endpoints. The staged training strategy further supports this adaptation: the encoder is initially frozen while downstream modules learn the OR-PAM feature space, and is subsequently fine-tuned for vascular refinement. The keypoint visualizations in Fig. 3 show that, after adaptation, detected keypoints concentrate on anatomically meaningful vascular landmarks, indicating that the encoder captures structure-aware vascular features rather than merely retaining natural-image priors.

This staged formulation also helps explain why the learning components remain effective with a limited number of OR-PAM training pairs. Stage 1 does not learn a full dense deformation field from scratch. It starts from pretrained keypoint and descriptor priors and adapts them to PA vascular images, where high spatial resolution and vascular bifurcations, crossings, and endpoints provide stable structural features compatible with natural-image feature learning. After PA-specific fine-tuning, Stage 1 supplies a large-scale TPS correction that brings the source and target vascular regions into approximate correspondence. The remaining problem for Stage 2 is therefore much narrower: it mainly consists of small local deformation and scan-direction-dependent PA signal-amplitude inconsistency within an already coarsely aligned vascular configuration. This decomposition reduces the need for Stage 2 to infer large global displacement from highly misaligned inputs, which helps explain the consistent performance observed on both DI and the unseen DII synthetic deformation validation set. In contrast, a single-stage unsupervised dense registration model may need to learn large-displacement correspondence and local refinement directly from intensity-similarity losses; when large deformation and PA amplitude inconsistency occur together, this optimization can become unstable even if the same model performs reasonably under small residual deformation.

The warping ablation clarifies the key design choice. Nearest-neighbor warping sacrifices a small amount of visual smoothness but avoids introducing interpolation-generated PA signal amplitudes. Compared with bilinear interpolation, the spatial penalty is limited: NCC decreases by 0.056 (6.39%), SSIM by 0.041 (4.85%), and PSNR by 0.07 dB (0.31%), while MSE is essentially unchanged. In contrast, PFr increases by 81.09 percentage points, from 16.74% to 97.83%, indicating stronger preservation of the measured PA signal-amplitude distribution. While bilinear interpolation can be useful for purely visual tasks, it is unsuitable for quantitative OR-PAM when downstream analysis depends on measured PA signal amplitudes. Even with nearest-neighbor warping, PFr remains below 100% due to frequency redistribution under spatial transformation. Thus, the method retains measured source PA amplitudes rather than guaranteeing exact histogram equality; measured distribution alteration is strongly reduced, but value distributions are not perfectly invariant.

The per-pair fine-tuning step reflects another deliberate trade-off. Consistent with prior work [34], [35], instance-specific optimization improves MSE (0.0144→0.0072), NCC (0.716→0.821), and SSIM (0.727→0.804), at the cost of increased runtime (3.33 s in fine-tuning mode vs. 0.12 s in direct-apply mode) and with a slight improvement in PFr (97.33%→97.83%). The fine-tuned model is used as the default because downstream tasks depend on both accurate spatial alignment and preservation of the measured PA signal-amplitude distribution. The direct-apply mode remains useful when speed is more important than peak accuracy. Thus, the full model represents a practical operating point rather than a universal optimum.

For the MNII metric, although VITAL achieves 0.876, well above the unregistered baseline (0.587) and all competitors, MNII is not a pure registration metric. It mixes two effects: spatial misalignment, which registration can fix, and PA amplitude inconsistency, which originates in the acquisition process and remains even after good alignment. As a result, MNII has a lower bound determined by residual stripe artifacts. Reaching the theoretical maximum would require additional amplitude normalization or hardware-level calibration. Within the current problem formulation, the substantial margin over the baselines indicates that VITAL captures most of the achievable geometric improvement.

Functional imaging is highly sensitive to registration failures, where even a single misaligned frame can distort sO${}_{2}$ evaluation, introduce artificial flow variations, or disrupt vessel continuity. The coarse-to-fine design relieves this risk: Stage 1 is robust to PA amplitude inconsistency through feature-space matching, while Stage 2 enforces locally consistent refinement via explicit regularization. Across DI and DII, the framework remained stable without manual retuning; in the representative inter-frame sequence, it also exhibited consistent temporal behavior under the validation settings examined here.

### 4.1 Limitations and future work

Despite the favorable performance demonstrated in this study, several limitations and practical considerations should be acknowledged. VITAL is primarily intended for imaging configurations that exhibit measurable bidirectional spatial mismatch and PA signal inconsistency. Imaging systems with sufficient intrinsic spatial alignment may not require a registration framework of comparable complexity. However, when residual acquisition-induced misalignment affects downstream quantitative analysis, accurate registration and preservation of measured PA signal distributions become important. Therefore, VITAL should be regarded as a targeted solution for quantitative functional OR-PAM systems in which such limitations remain a practical challenge.

The current Stage 2 model is trained on the same dataset as Stage 1, and its generalization would benefit from larger and more diverse data. This limitation is particularly relevant for the dense Stage 2 refinement module, which has higher capacity than the explicit keypoint/TPS stage. The architecture reduces the effective deformation capacity by combining pretrained keypoint and matching priors, an explicit TPS transformation in Stage 1, and residual local refinement with smoothness and Jacobian constraints in Stage 2. The DI split evaluates cross-time-point generalization across held-out acquisition time points within the acquired dynamic imaging distribution. The DII synthetic validation provides complementary evidence under controlled affine-elastic deformation, and DIII/DIV mouse ear-vessel experiments provide additional external validation across different vascular morphology and imaging conditions, while larger multi-session and multi-animal validation remains an important future direction. The downstream sO${}_{2}$ and $v_{\text{flow}}$ analyses are demonstrated on a representative 47-frame oxygen-challenge sequence, and broader validation across additional complete functional sequences remains future work. In addition, the method is currently limited to 2D maximum amplitude projections (MAP) of PA signals, and extension to 3D raw PA data remains an important direction for future work.

Potential failure cases may occur when the vascular signal is weak, the signal-to-noise ratio is low, or the field of view contains few stable landmarks such as bifurcations, crossings, and endpoints. Repetitive parallel vessel patterns, severe out-of-plane motion, or deformation far outside the training distribution may also reduce the reliability of Stage 1 matching. Because Stage 2 is designed as a residual local refinement module, it cannot reliably recover a globally incorrect Stage 1 correspondence. For practical use, such cases can be screened using quality-control indicators including the number of detected keypoints, the number of accepted matches, LightGlue matching confidence, TPS deformation magnitude, Jacobian folding rate, residual spatial metrics, MNII, and PFr/value-frequency scatter behavior.

Nearest-neighbor warping avoids interpolation-generated PA signal amplitudes but may introduce visible aliasing at vessel boundaries. It can also duplicate, omit, or reassign source pixels depending on the deformation field; therefore, it retains measured source PA amplitudes rather than guaranteeing quantitatively correct anatomical PA measurements by itself. More advanced schemes, such as quantization-aware or lookup-table-based methods that constrain outputs to the source histogram while improving smoothness, may better balance geometric accuracy and signal fidelity.

## 5 Conclusion

This work presents VITAL, an interpretable two-stage registration framework for high-speed OR-PAM. By explicitly decoupling geometric alignment from preservation of discrete source PA amplitude distributions, VITAL combines keypoint-driven global registration with topology-preserving dense refinement to achieve robust performance under PA amplitude inconsistency and spatial misalignment. A per-pair fine-tuning step further adapts the deformation field at inference. The proposed warping strategy avoids interpolation-generated PA signal amplitudes, helping reduce interpolation-induced signal distortion in multi-wavelength sO${}_{2}$, blood-flow-speed estimation, and vascular-continuity assessment. These findings establish PSAP registration as an important requirement for quantitative functional OR-PAM, advancing a principled pipeline from raw acquisition to reliable hemodynamic interpretation.

## CRediT authorship contribution statement

**Shuocheng Qi:** Writing – original draft, Visualization, Validation, Software, Methodology, Formal analysis, Conceptualization. **Mingxuan Wang:** Visualization, Software. **Yachao Zhang:** Investigation, Data curation. **Lidai Wang:** Writing – review & editing, Supervision, Resources. **Chao Liu:** Writing – review & editing, Validation, Supervision, Resources, Project administration, Methodology, Funding acquisition, Data curation, Conceptualization.

## Declaration of competing interest

The authors declare that they have no known competing financial interests or personal relationships that could have appeared to influence the work reported in this paper.

## Data Availability

Data will be made available on request.

## Appendix A Supplementary material

Supplementary Material: Additional dataset descriptions and validation results are provided in Supplementary Notes S1–S7, Figures S1–S4, and Tables S1–S4.

Supplementary Video S1: Temporal dynamics of sO${}_{2}$ and $v_{\text{flow}}$ across the full imaging sequence, covering baseline, N${}_{2}$ challenge (hypoxia), and air recovery after VITAL registration.

MMC S1 Supplementary material containing additional methods, figures, tables, and experimental results. MMC S1
