# Supplementary material for: VITAL: Value-Invariant Transformation and Alignment Learning for quantitative photoacoustic microscopy
Source: Photoacoustics. 2026 Jun 8;50:100845. doi: 10.1016/j.pacs.2026.100845 (PMC13273772; doi:10.1016/j.pacs.2026.100845)
Supplement: MMC S1 — Supplementary material containing additional methods, figures, tables, and experimental results. [file mmc1.pdf]

Supplementary Material for “VITAL: Value-Invariant Transformation and Alignment Learning for Quantitative Photoacoustic Microscopy”

Shuocheng Qi[a,b,c], Mingxuan Wang[f], Yachao Zhang[g,h], Lidai Wang[d,e,*], Chao Liu[a,b,*]

[a]Digital Medical Research Center, School of Basic Medical Sciences, Fudan University, Shanghai, China

[b]Shanghai Key Laboratory of Medical Imaging Computing and Computer Assisted Intervention, Shanghai, China

[c]School of Intelligent Equipment, Shandong University of Science and Technology, Shandong, China

[d]Department of Biomedical Engineering, City University of Hong Kong, Kowloon, Hong Kong SAR, China

[e]Shenzhen Research Institute, City University of Hong Kong, Shenzhen, China

[f]School of Information Science and Technology, Guangdong University of Foreign Studies, Guangzhou, China

[g]The Suzhou Institute of Biomedical Engineering and Technology, Chinese Academy of Sciences, Suzhou 215163, China

[h]The School of Biomedical Engineering (Suzhou), Division of Life Sciences and Medicine, University of Science and Technology of China, Hefei, 230026, China

[*]Corresponding Authors: Lidai Wang (lidawang@cityu.edu.hk) & Chao Liu (chaoliu@fudan.edu.cn)

Contents

- Supplementary Note S1. Dataset scope, split level, and validation setting.
- Supplementary Note S2. Baseline protocol and final resampling evaluation.
- Supplementary Note S3. PA signal-amplitude distribution fidelity rate (PFr) definition and nearest-neighbor final resampling.
- Supplementary Note S4. Dataset III (DIII) independent mouse-ear external validation.
- Supplementary Note S5. Dataset IV (DIV) independent mouse-ear external validation.
- Supplementary Note S6. Dataset II (DII) synthetic known-deformation validation.
- Supplementary Note S7. Inter-frame registration and functional-analysis scope.
- Supplementary Tables S1-S4 and Figures S1-S4.
- Supplementary Video S1 description.

Supplementary Note S1. Dataset Scope, Split Level, and Validation Setting

The main manuscript evaluates VITAL on real optical-resolution photoacoustic microscopy (OR-PAM) odd-even registration and additional validation settings. The original Dataset I (DI) development split is a pooled image-pair-level split. The results from this split therefore report performance on held-out image pairs under related acquisition conditions, while cross-animal and cross-system generalization can be examined in future studies.

Additional validation includes Dataset II (DII) synthetic known-deformation data and two independent mouse ear-vessel OR-PAM sequences, Dataset III (DIII; Mouse Ear I) and Dataset IV (DIV; Mouse Ear II), covering multiple external vascular cases. DIII is a continuous 15-frame mouse ear-vessel measurement sequence. DIV is a separate continuous 90-frame mouse ear-vessel measurement sequence that was acquired to further test generalization beyond the shorter DIII sequence. The synthetic deformation experiments provide known transformations, whereas the odd-line target in real OR-PAM serves as a fixed reference rather than an independent anatomical ground truth.

Table S1. Dataset use and interpretation scope.

Dataset	Type	Use	Interpretation scope
Dataset I (DI)	Real *in vivo* mouse-brain OR-PAM odd-even pairs	Main held-out pair-level brain test set	Supports intra-frame registration under the manuscript brain-imaging acquisition setting
Dataset II (DII)	Controlled synthetic deformation data	Known-deformation validation setting	Separates geometric recovery from odd-line reference limitations
Dataset III (DIII; Mouse Ear I)	Continuous 15-frame mouse ear-vessel OR-PAM validation sequence	Independent external generalization experiment	Evaluates transfer to a new vascular morphology and imaging scenario using a short continuous ear-vessel sequence
Dataset IV (DIV; Mouse Ear II)	Separate continuous 90-frame mouse ear-vessel OR-PAM validation sequence	Independent external generalization experiment	Further evaluates generalization over a longer continuous ear-vessel sequence beyond the shorter DIII acquisition
Inter-frame sequences	Dynamic OR-PAM frame series, including Brain-A, Brain-B, and Brain-C	Reference-frame alignment after intra-frame correction	Provides representative temporal analysis complementary to multi-animal functional validation

Supplementary Note S2. Baseline Protocol and Final Resampling Evaluation

Baseline comparisons are organized in two layers. First, deformation quality is evaluated by spatial metrics such as mean squared error (MSE), normalized cross-correlation (NCC), structural similarity index measure (SSIM), peak signal-to-noise ratio (PSNR), visual vessel continuity, and synthetic deformation controls. Second, final resampling fidelity is evaluated by photoacoustic (PA) signal-amplitude distribution fidelity rate (PFr) and value-frequency analysis. This separation accounts for the effect of interpolation choice on PFr independently of the estimated deformation field.

Learning-based baselines with nearest-neighbor (NN) final resampling are included where available. These variants keep the final resampling mode comparable while preserving each method-specific deformation-estimation procedure. Method-specific default output denotes the registered image produced by each baseline implementation using its standard resampling setting.

Table S2. Registration method protocol summary.

**Method**	**Role**	**Training / adaptation**	**Final resampling**	**Per-pair optimization**
Scale-Invariant Feature Transform (SIFT)	Classical keypoint baseline	No training	Method-specific default output	No
Optical flow	Intensity-driven dense motion	No training	Method-specific default output	No
Demons	Classical deformable registration	No training	Method-specific default output	No
Symmetric Normalization (SyN)	Symmetric normalization	No training	Method-specific default output	No
VoxelMorph	Learning-based dense registration	Same development data; reported one-shot and fine-tuned variants	Original and nearest-neighbor (NN)-controlled variants	Yes, where labeled
TransMorph	Transformer-based dense registration	Same development data; reported one-shot and fine-tuned variants	Original and nearest-neighbor (NN)-controlled variants	Yes, where labeled
Value-Invariant Transformation and Alignment Learning (VITAL)	Keypoint/thin-plate spline (TPS) global stage plus constrained dense residual refinement	Same development data; direct and per-pair optimized modes	Nearest-neighbor final warp	Yes, where labeled

Supplementary Note S3. PFr Definition and Nearest-Neighbor Final Resampling

PFr is defined in the main manuscript as a histogram-intersection metric for preservation of the measured PA signal-amplitude distribution during registration. PA signal amplitudes and derived oxygen saturation (sO_2) values are uniformly quantized to an integer range of [0, 10000], and value-frequency histograms are compared between the source region of interest (ROI) and the registered output inside the target ROI, with background zeros excluded. In the manuscript, the target ROI is the method-independent Otsu-derived vessel ROI generated from the fixed/target image, and the source ROI is generated from the source image using the same Otsu-thresholding and post-processing procedure. Thus, PFr evaluates how much the measured PA signal-amplitude distribution is preserved after registration, including the effects of interpolation-generated PA signal amplitudes, loss of source-value bins, and redistribution of value frequencies. It complements spatial metrics such as MSE, NCC, SSIM, and PSNR rather than replacing them; high PFr should be interpreted together with spatial alignment metrics and visual assessment. When spatial metrics are strong and PFr is also high, the result supports two complementary interpretations: the registered vessels exhibit improved anatomical correspondence, and the PA signal-amplitude distribution within the target registration region has not been substantially altered by method-induced misplacement of signals from other regions or by new amplitudes introduced through interpolation during final resampling. Thus, the combined evidence of high spatial alignment and high PFr supports both improved anatomical correspondence and reduced alteration of measured PA signal distributions, providing a stronger basis for downstream functional analysis.

Nearest-neighbor final resampling is used in VITAL to avoid interpolation-generated PA signal amplitudes. Each output pixel is sampled from an existing measured source PA amplitude rather than synthesized by averaging neighboring pixels. However, nearest-neighbor warping does not guarantee exact histogram equality or quantitative anatomical correctness by itself: depending on the deformation field, it can duplicate, omit, or reassign source pixels, and spatial resampling can still redistribute the frequency of existing amplitudes. Therefore, the manuscript describes nearest-neighbor warping as retaining measured source PA amplitudes and supporting preservation of discrete source PA amplitude distributions, rather than as guaranteeing invariant histograms. This distinction is important for quantitative OR-PAM, because downstream sO_2 and blood flow speed (v_{flow}) analyses depend on measured multi-wavelength or temporal PA signal amplitudes. Bilinear or bicubic interpolation may improve visual smoothness and some image-similarity scores, but it can generate PA signal amplitudes that were not directly measured and perturb the spectral or temporal relationships used for functional estimation.

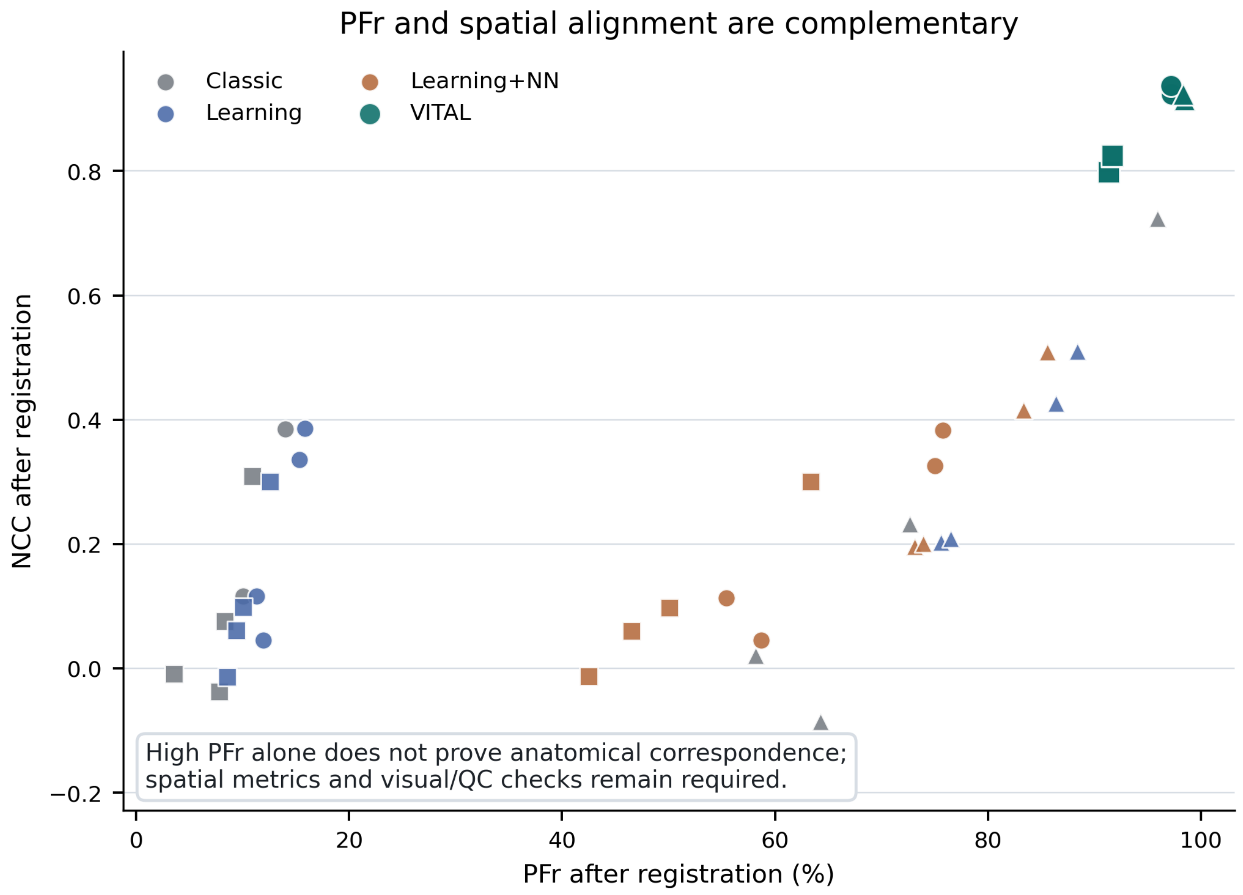

Figure S1. Relationship between PA signal-amplitude distribution fidelity and spatial alignment after registration. The horizontal axis shows PFr after registration, and the vertical axis shows NCC after registration. Marker colors group method families: classic methods, learning-based methods, learning-based methods with nearest-neighbor final resampling, and VITAL. Different marker shapes distinguish the evaluated validation subsets and operating points. The upper-right region combines higher preservation of the measured PA signal-amplitude distribution with higher anatomical similarity. The plot presents PFr together with NCC so that PA signal-amplitude distribution preservation and anatomical similarity can be assessed jointly.

Supplementary Note S4. DIII Independent Mouse-Ear External Validation

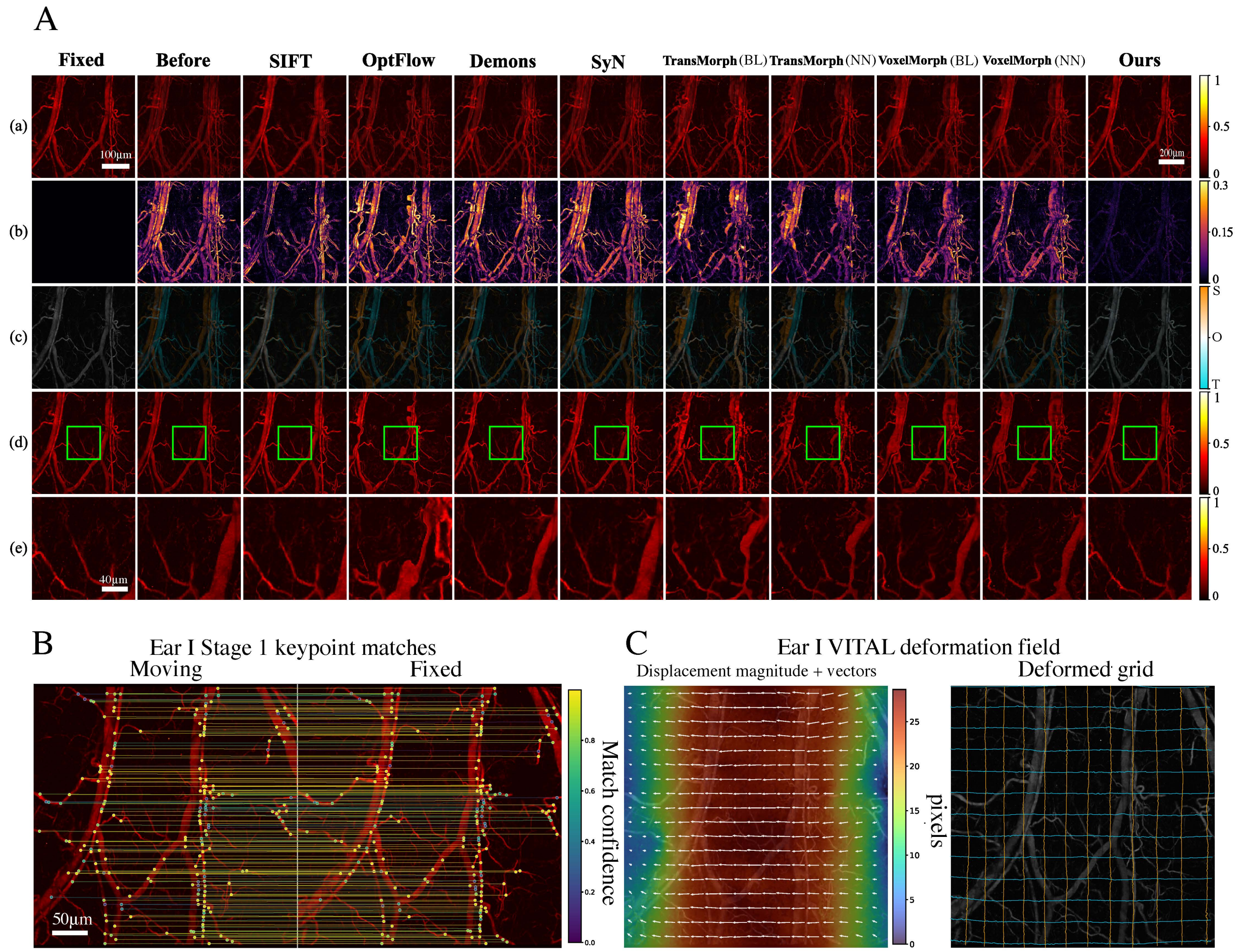

Figure S2. DIII (Mouse Ear I) independent external validation on a continuous 15-frame mouse ear-vessel OR-PAM measurement sequence. (A) Registration-performance comparison shown in the same layout as the main intra-frame registration figure. (a) Full field-of-view images of the target image (odd lines only), the unregistered odd-even interleaved image, and the registered results obtained by different methods. (b) and (c) show absolute difference maps and color overlays with respect to the target odd-line image, including the self-baseline and the corresponding even-line images from the unregistered and registered results. Lower residual intensity and stronger structural overlap indicate better alignment. (d) From left to right: the target odd-line image, the source even-line image before registration, and the registered even-line images produced by different methods. (e) Magnified views of the region highlighted by the green box in (d), showing vascular continuity and boundary artifacts across methods. From top to bottom, the colorbars represent normalized PA signal intensity (0–1) for (a), (d), and (e); absolute PA signal-intensity difference (0–0.3) for (b); and source–target overlay categories (S: source, O: overlap, T: target) for (c). In the method labels, bilinear (BL) denotes the original bilinear final-resampling output, whereas nearest-neighbor (NN) denotes the nearest-neighbor final-resampling control applied to the same predicted deformation field. (B) Stage-1 feature matching result. SuperPoint is used to detect vascular keypoints, and LightGlue is used to establish the matched keypoint connections used for TPS-based coarse alignment. (C) VITAL deformation-field visualization, including the estimated deformation field and the corresponding deformation grid.

Dataset III (DIII; Mouse Ear I) is a continuous 15-frame mouse ear-vessel OR-PAM measurement sequence used as an independent external validation case outside the main mouse-brain Dataset I (DI) test set. The same registration workflow was applied to this external vascular sequence to evaluate whether

the proposed method can handle a different vascular morphology and imaging condition. Figure S2 summarizes the experiment from three complementary views: the final registration comparison, the Stage-1 vascular landmark correspondences used for thin-plate spline (TPS)-based coarse alignment, and the VITAL deformation field. The improved structural overlap, interpretable landmark matching, and spatially coherent deformation field indicate that VITAL can recover meaningful vessel correspondence beyond the primary brain odd-even evaluation setting.

Supplementary Note S5. DIV Independent Mouse-Ear External Validation

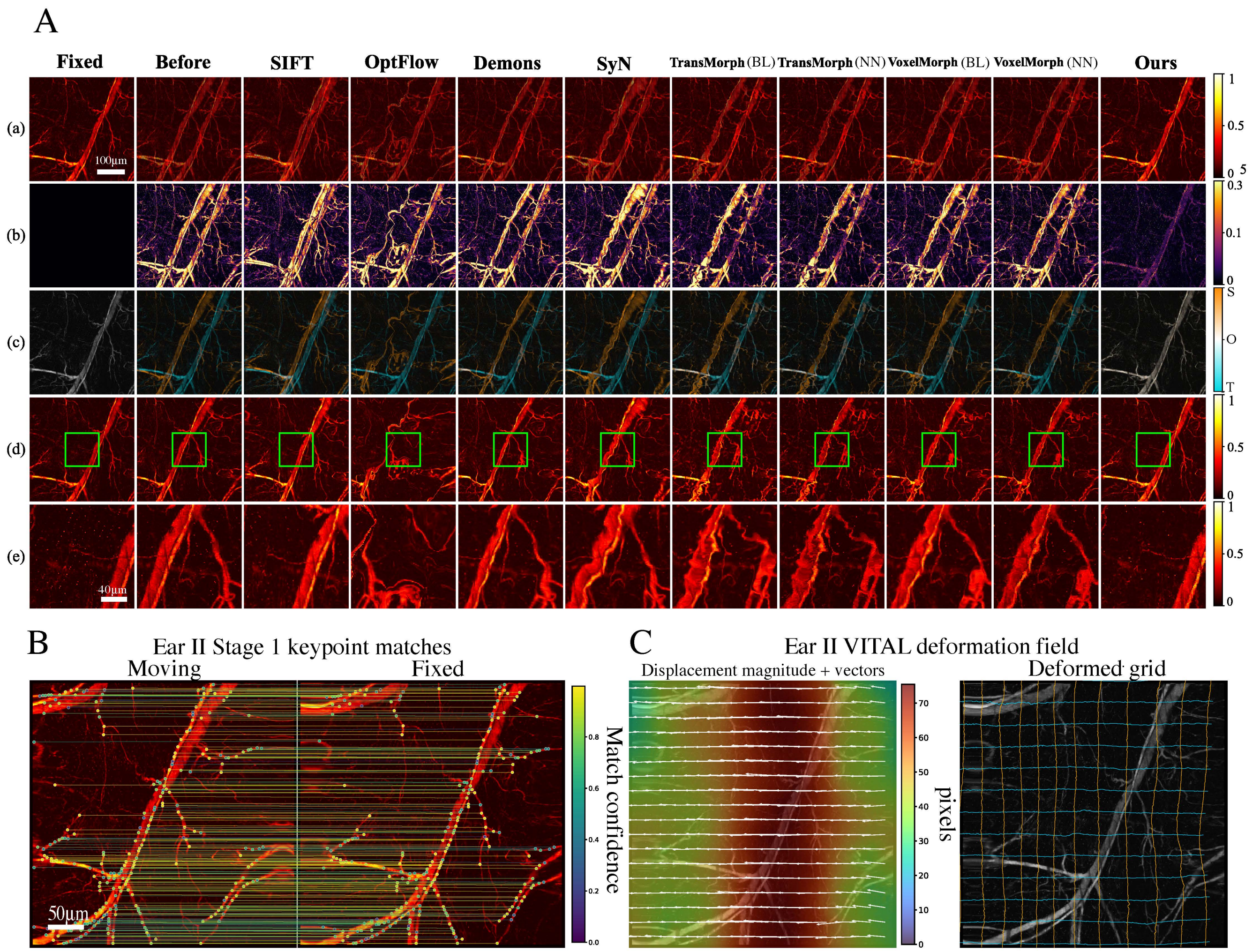

Figure S3. DIV (Mouse Ear II) independent external validation on a separate continuous 90-frame mouse ear-vessel OR-PAM measurement sequence acquired to further test generalization beyond the shorter DIII sequence. (A) Registration-performance comparison shown in the same layout as the main intra-frame registration figure. (a) Full field-of-view images of the target image (odd lines only), the unregistered odd-even interleaved image, and the registered results obtained by different methods. (b) and (c) show absolute difference maps and color overlays with respect to the target odd-line image, including the self-baseline and the corresponding even-line images from the unregistered and registered results. Lower residual intensity and stronger structural overlap indicate better alignment. (d) From left to right: the target odd-line image, the source even-line image before registration, and the registered even-line images produced by different methods. (e) Magnified views of the region highlighted by the green box in (d), showing vascular continuity and boundary artifacts across methods. From top to bottom, the colorbars represent normalized PA signal intensity (0–1) for (a), (d), and (e); absolute PA signal-intensity difference (0–0.3) for (b); and source–target overlay categories (S: source, O: overlap, T: target) for (c). In the method labels, bilinear (BL) denotes the original bilinear final-resampling output, whereas nearest-neighbor (NN) denotes the nearest-neighbor final-resampling control applied to the same predicted deformation field. (B) Stage-1 feature matching result. SuperPoint is used to detect vascular keypoints, and LightGlue is used to establish the matched keypoint connections used for TPS-based coarse alignment. (C) VITAL deformation-field visualization, including the estimated deformation field and the corresponding deformation grid.

Dataset IV (DIV; Mouse Ear II) is a separate continuous 90-frame mouse ear-vessel OR-PAM measurement sequence acquired as a second independent external validation case. Because DIII contains

only 15 consecutive frames, DIV was specifically included to test whether the registration performance remains consistent over a longer continuous ear-vessel sequence and is not limited to the shorter DIII acquisition. Figure S3 presents the final registration comparison, the Stage-1 landmark matching result, and the estimated VITAL deformation field. The agreement between DIII and DIV supports that the observed improvement is not specific to a single external example and provides additional evidence for generalization across independent vascular images and longer continuous measurements.

Supplementary Note S6. DII Synthetic Known-Deformation Validation

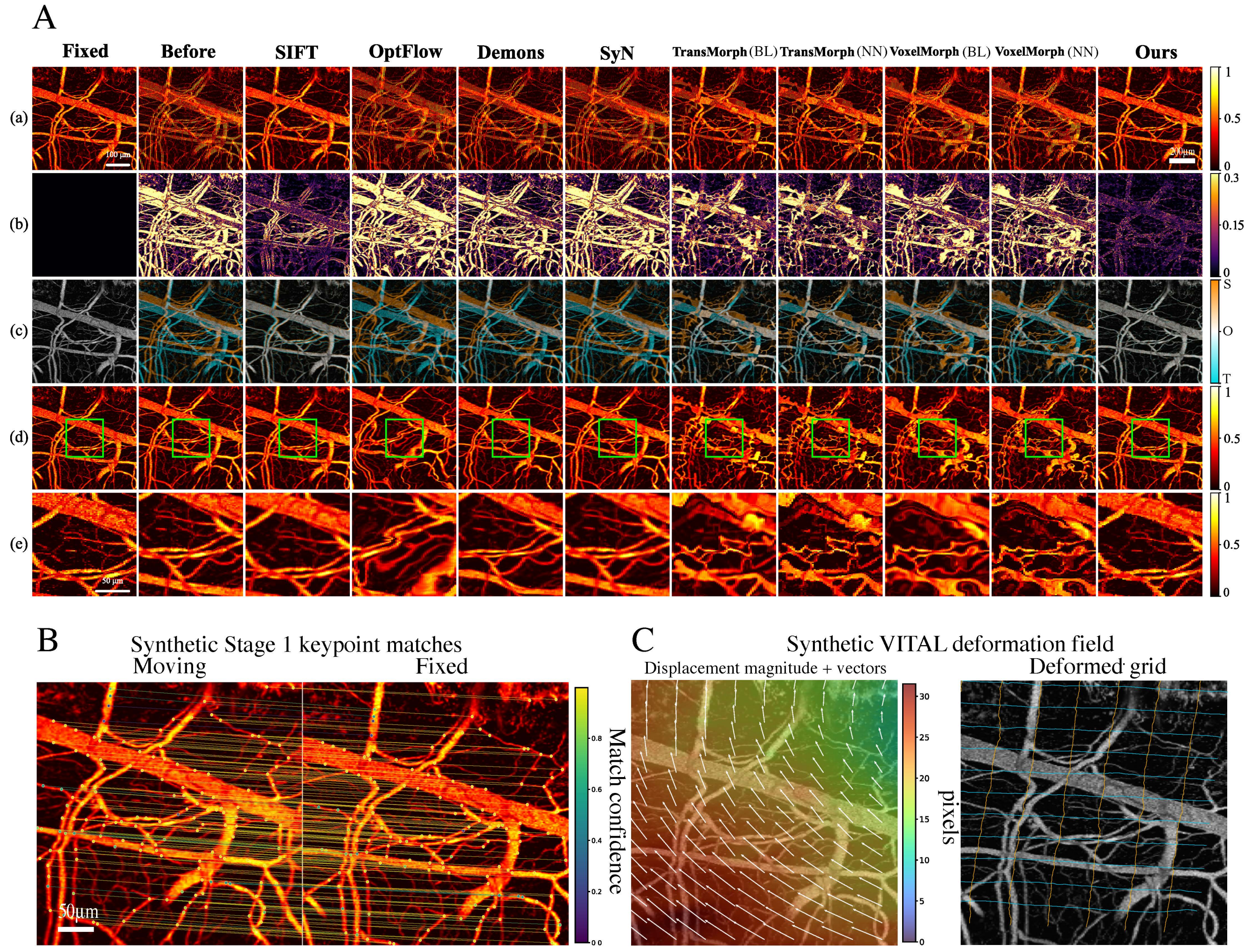

Figure S4. DII synthetic known-deformation validation under controlled affine-elastic deformation. The synthetic moving images were generated from real OR-PAM vascular images using random translation up to 28 pixels, random rotation up to 5°, no global scale change, and a smooth elastic random field generated on a 7 × 5 grid with elastic scale 1.0; the random seed was fixed at 443. (A) Registration-performance comparison shown in the same layout as the main intra-frame registration figure. (a) Full field-of-view images of the target image (odd lines only), the unregistered odd-even interleaved image, and the registered results obtained by different methods. (b) and (c) show absolute difference maps and color overlays with respect to the target odd-line image, including the self-baseline and the corresponding even-line images from the unregistered and registered results. Lower residual intensity and stronger structural overlap indicate better alignment. (d) From left to right: the target odd-line image, the source even-line image before registration, and the registered even-line images produced by different methods. (e) Magnified views of the region highlighted by the green box in (d), showing vascular continuity and boundary artifacts across methods. From top to bottom, the colorbars represent normalized PA signal intensity (0–1) for (a), (d), and (e); absolute PA signal-intensity difference (0–0.3) for (b); and source–target overlay categories (S: source, O: overlap, T: target) for (c). In the method labels, bilinear (BL) denotes the original bilinear final-resampling output, whereas nearest-neighbor (NN) denotes the nearest-neighbor final-resampling control applied to the same predicted deformation field. (B) Stage-1 feature matching result. SuperPoint is used to detect vascular keypoints, and LightGlue is used to establish the matched keypoint connections used for TPS-based coarse alignment. (C) VITAL deformation-field visualization, including the estimated deformation field and the corresponding deformation grid.

The Dataset II (DII) synthetic known-deformation validation was constructed from real OR-PAM vascular images to evaluate registration under a controlled affine-elastic deformation. Synthetic moving images were generated from fixed images using the same deformation across wavelength bands to preserve inter-band correspondence. The imposed deformation consisted of random translation up to 28 pixels, random rotation up to 5°, no global scale change, and a smooth elastic random field generated on a 7×5 grid with elastic scale 1.0; the random seed was fixed at 443. The moving-to-fixed displacement field and valid mask were saved for each pair. DII contains 48 synthetic odd-even pairs and was used only for validation; no model was trained on DII, and the learning-based models trained on the DI training split were directly applied to this unseen deformation setting. This design tests whether each method can generalize beyond the limited DI training distribution. Under this larger controlled deformation, the two single-stage dense learning baselines, VoxelMorph and TransMorph, show reduced generalization performance in the small-sample setting because they must infer large displacement and local refinement directly from intensity-similarity losses. In contrast, VITAL benefits from Stage 1 global TPS alignment driven by SuperPoint-LightGlue vascular correspondences. This coarse global correction brings the source and target vascular regions into approximate correspondence before Stage 2 refinement, effectively moving the residual problem back into a more familiar local-alignment regime. Figure S4 therefore provides controlled evidence that the staged design improves generalization under unseen deformation while maintaining PA signal-amplitude distribution fidelity.

Supplementary Note S7. Inter-Frame Registration and Functional-Analysis Scope

The main manuscript reports inter-frame registration and downstream functional analysis on one representative complete 47-frame oxygen-challenge sequence selected from the four independent mouse-brain acquisition sessions described in the Dataset section. The remaining three mouse-brain acquisition sessions are reported here as Brain-A, Brain-B, and Brain-C to provide additional inter-frame registration validation beyond the sequence shown in the main text. For each sequence, intra-frame correction was first applied to each frame, and the corrected full-resolution frames were then aligned to the first frame using the same pretrained two-stage model without additional sequence-specific retraining. Stage 1 estimates a global thin-plate spline (TPS) field from vascular keypoint correspondences between each dynamic frame and the reference frame, and Stage 2 refines the residual mismatch with a dense deformation field. Because inter-frame motion is primarily rigid or low-order after intra-frame correction, this experiment evaluates whether the pretrained model can transfer to temporal frame alignment across additional brain acquisition sessions. Brain-A, Brain-B, and Brain-C therefore complement the main 47-frame functional sequence by showing that the inter-frame registration performance is consistent across the other three brain sessions, while DIII and DIV further test external mouse ear-vessel generalization.

Supplementary Video S1 provides a full-sequence visualization of this oxygen-challenge experiment across baseline, nitrogen gas (N_2) challenge, and air recovery.

Table S3. Inter-frame registration metrics for additional mouse-brain acquisition sessions and independent mouse ear-vessel validation cases.

Dataset	MSE before	MSE Stage 1	MSE after	NCC before	NCC Stage 1	NCC after
Brain-A	0.049	0.022	0.016	0.103	0.689	0.816
Brain-B	0.023	0.009	0.007	0.112	0.661	0.791
Brain-C	0.087	0.031	0.021	-0.231	0.569	0.714
DIII (Mouse Ear I)	0.005	0.006	0.003	0.654	0.577	0.772
DIV (Mouse Ear II)	0.021	0.014	0.009	0.513	0.708	0.839

Supplementary Tables

Table S4 summarizes the benchmark results across available methods and datasets, allowing comparison of trends across validation settings. Abbreviations used in the benchmark tables are as follows: FT, fine-tuned with per-pair optimization; NN, nearest-neighbor final resampling; MSE, mean squared error; NCC, normalized cross-correlation; SSIM, structural similarity index measure; PSNR, peak signal-to-noise ratio; PFr, PA signal-amplitude distribution fidelity rate; and std, standard deviation.

Table S4. Full benchmark summary across available datasets. FT denotes fine-tuned with per-pair optimization.

Method	Dataset	MSE mean	MSE std	NCC mean	NCC std	SSIM mean	SSIM std	PSNR mean	PSNR std	PFr mean	PFr std
Demons	DIII (Mouse Ear I)	0.0071	0.0016	0.117	0.059	0.362	0.028	21.61	0.92	10.06	1.03
Optical flow	DIII (Mouse Ear I)	0.0084	0.0019	-0.124	0.022	0.287	0.030	20.83	0.85	5.08	0.86
SIFT	DIII (Mouse Ear I)	0.0055	0.0019	0.385	0.089	0.402	0.058	22.87	1.37	14.00	1.49
SyN	DIII (Mouse Ear I)	0.0102	0.0016	-0.172	0.060	0.229	0.023	19.95	0.69	8.49	0.82
TransMorph	DIII (Mouse Ear I)	0.0093	0.0026	0.045	0.046	0.315	0.033	20.46	1.14	11.93	0.74
TransMorph -FT	DIII (Mouse Ear I)	0.0068	0.0036	0.386	0.073	0.540	0.046	22.10	1.70	15.84	0.83
TransMorph -FT-NN	DIII (Mouse Ear I)	0.0050	0.0012	0.383	0.070	0.547	0.026	23.16	0.93	75.76	2.55
TransMorph -NN	DIII (Mouse Ear I)	0.0071	0.0011	0.045	0.045	0.328	0.020	21.53	0.64	58.72	2.90
VITAL	DIII (Mouse Ear I)	0.0007	0.0002	0.923	0.020	0.861	0.026	31.89	1.24	97.24	0.74
VITAL-FT	DIII (Mouse Ear I)	0.0011	0.0019	0.937	0.016	0.864	0.069	31.71	3.14	97.17	0.90
VoxelMorph	DIII (Mouse Ear I)	0.0075	0.0017	0.116	0.056	0.350	0.025	21.32	0.88	11.31	0.83
VoxelMorph -FT	DIII (Mouse Ear I)	0.0057	0.0009	0.336	0.047	0.462	0.018	22.53	0.66	15.32	0.87
VoxelMorph -FT-NN	DIII (Mouse Ear I)	0.0049	0.0007	0.326	0.045	0.458	0.018	23.10	0.58	75.03	3.23
VoxelMorph -NN	DIII (Mouse Ear I)	0.0064	0.0010	0.113	0.055	0.354	0.021	21.96	0.66	55.45	3.68
Demons	DIV (Mouse Ear II)	0.0314	0.0091	0.076	0.035	0.204	0.027	15.24	1.40	8.33	0.59
Optical flow	DIV (Mouse Ear II)	0.0301	0.0103	-0.008	0.016	0.190	0.035	15.48	1.53	3.54	0.48
SIFT	DIV (Mouse Ear II)	0.0255	0.0075	0.309	0.106	0.219	0.040	16.12	1.33	10.93	1.86
SyN	DIV (Mouse Ear II)	0.0386	0.0102	-0.038	0.048	0.140	0.026	14.31	1.27	7.79	0.61
TransMorph	DIV (Mouse Ear II)	0.0346	0.0095	0.061	0.034	0.187	0.027	14.79	1.32	9.42	0.57

TransMorph -FT	DIV (Mouse Ear II)	0.0267	0.0074	0.300	0.061	0.379	0.036	15.93	1.36	12.58	0.72
TransMorph -FT-NN	DIV (Mouse Ear II)	0.0256	0.0075	0.300	0.059	0.363	0.037	16.14	1.44	63.36	3.05
TransMorph -NN	DIV (Mouse Ear II)	0.0321	0.0102	0.060	0.033	0.181	0.031	15.18	1.54	46.54	2.54
VITAL	DIV (Mouse Ear II)	0.0093	0.0056	0.799	0.097	0.653	0.085	20.84	2.04	91.32	5.29
VITAL-FT	DIV (Mouse Ear II)	0.0084	0.0054	0.824	0.092	0.697	0.085	21.33	2.15	91.70	4.93
VoxelMorph	DIV (Mouse Ear II)	0.0381	0.0103	-0.014	0.040	0.163	0.024	14.38	1.33	8.59	0.59
VoxelMorph -FT	DIV (Mouse Ear II)	0.0326	0.0086	0.099	0.051	0.224	0.025	15.04	1.25	10.08	0.70
VoxelMorph -FT-NN	DIV (Mouse Ear II)	0.0306	0.0094	0.098	0.049	0.215	0.029	15.38	1.50	50.11	3.01
VoxelMorph -NN	DIV (Mouse Ear II)	0.0346	0.0108	-0.013	0.039	0.159	0.028	14.85	1.52	42.53	2.67
Demons	DII (Synthetic)	0.0572	0.0147	0.232	0.180	0.233	0.114	12.60	1.31	72.64	5.36
Optical flow	DII (Synthetic)	0.0749	0.0081	0.021	0.068	0.045	0.016	11.28	0.48	58.21	4.61
SIFT	DII (Synthetic)	0.0212	0.0044	0.724	0.057	0.500	0.085	16.83	0.92	95.95	1.78
SyN	DII (Synthetic)	0.0831	0.0111	-0.085	0.100	0.035	0.027	10.84	0.61	64.27	4.33
TransMorph	DII (Synthetic)	0.0615	0.0124	0.202	0.145	0.177	0.062	12.21	0.96	75.57	4.21
TransMorph -FT	DII (Synthetic)	0.0379	0.0089	0.510	0.113	0.466	0.075	14.36	1.16	88.41	2.60
TransMorph -FT-NN	DII (Synthetic)	0.0379	0.0089	0.509	0.109	0.456	0.079	14.35	1.15	85.57	4.86
TransMorph -NN	DII (Synthetic)	0.0619	0.0120	0.196	0.138	0.166	0.060	12.17	0.90	73.10	5.36
VITAL	DII (Synthetic)	0.0066	0.0014	0.915	0.018	0.826	0.024	21.90	0.92	98.44	0.67
VITAL-FT	DII (Synthetic)	0.0060	0.0012	0.923	0.015	0.841	0.021	22.34	0.88	98.33	0.66
VoxelMorph	DII (Synthetic)	0.0613	0.0172	0.209	0.206	0.185	0.103	12.34	1.46	76.50	5.15
VoxelMorph -FT	DII (Synthetic)	0.0441	0.0172	0.426	0.222	0.351	0.176	14.09	2.55	86.39	4.52
VoxelMorph -FT-NN	DII (Synthetic)	0.0446	0.0173	0.416	0.219	0.341	0.184	14.05	2.59	83.36	7.96
VoxelMorph -NN	DII (Synthetic)	0.0612	0.0163	0.201	0.197	0.174	0.100	12.32	1.36	73.95	6.51

Supplementary Video

Supplementary Video S1. Temporal dynamics of sO_2 and v_{flow} across the oxygen-challenge sequence after VITAL registration. The video provides a representative dynamic visualization covering baseline, nitrogen gas (N_2) challenge, and air recovery.
